# Au-activated N motifs in non-coherent cupric porphyrin metal organic frameworks for promoting and stabilizing ethylene production

**DOI:** 10.1038/s41467-021-27768-6

**Published:** 2022-01-17

**Authors:** Xulan Xie, Xiang Zhang, Miao Xie, Likun Xiong, Hao Sun, Yongtao Lu, Qiaoqiao Mu, Mark H. Rummeli, Jiabin Xu, Shuo Li, Jun Zhong, Zhao Deng, Bingyun Ma, Tao Cheng, William A. Goddard, Yang Peng

**Affiliations:** 1 Soochow Institute for Energy and Materials Innovations, College of Energy, Soochow University, Suzhou, 215006 China; 2 Key Laboratory of Advanced Carbon Materials and Wearable Energy Technologies of Jiangsu Province, Suzhou, 215006 China; 3 Institute of Functional Nano and Soft Materials (FUNSOM), Soochow University, Suzhou, 215123 China; 4 School of Chemical and Environmental Engineering, Shanghai Institute of Technology, Shanghai, 201418 China; 5 Jiangsu Engineering Laboratory of New Materials for Sewage Treatment and Recycling, Suzhou, 215123 China; 6 Materials and Process Simulation Center, Department of Chemistry, California Institute of Technology, Pasadena, CA 91125 United States

**Keywords:** Energy, Electrocatalysis, Metal-organic frameworks

## Abstract

Direct implementation of metal-organic frameworks as the catalyst for CO_2_ electroreduction has been challenging due to issues such as poor conductivity, stability, and limited > 2e^−^ products. In this study, Au nanoneedles are impregnated into a cupric porphyrin-based metal-organic framework by exploiting ligand carboxylates as the Au^3+^ -reducing agent, simultaneously cleaving the ligand-node linkage. Surprisingly, despite the lack of a coherent structure, the Au-inserted framework affords a superb ethylene selectivity up to 52.5% in Faradaic efficiency, ranking among the best for metal-organic frameworks reported in the literature. Through operando X-ray, infrared spectroscopies and density functional theory calculations, the enhanced ethylene selectivity is attributed to Au-activated nitrogen motifs in coordination with the Cu centers for C-C coupling at the metalloporphyrin sites. Furthermore, the Au-inserted catalyst demonstrates both improved structural and catalytic stability, ascribed to the altered charge conduction path that bypasses the incoherent framework. This study underlines the modulation of reticular metalloporphyrin structure by metal impregnation for steering the CO_2_ reduction reaction pathway.

## Abstract (web summary)

Metal-organic frameworks are promising catalysts for CO_2_ electroreduction, yet limited by their poor conductivity and stability. Here, Au nanoneedles are inserted into the metalloporphyrin framework to activate C-C coupling and stabilize the structure for much enhanced ethylene production.

## Introduction

Growing concern on the escalating anthropogenic carbon footprint urges all countries around the world to chart their carbon neutrality plans, in which using renewable electricity to convert CO_2_ into value-added chemicals is highly tempting and promising^1–3^. However, the high energy barrier for CO_2_ activation and the linear scaling relation for binding of intermediates constitute two intrinsic challenges for achieving high energy efficiency and product selectivity. This necessitates the development of high-performance electrocatalysts for CO_2_ reduction reactions (CO_2_RR)^4–6^. In this context, tremendous efforts have been devoted to the design and engineering of a wide category of CO_2_RR catalysts targeted for selectively producing C_1_ small molecules^7–9^, C_2+_ hydrocarbons^10,11^, and oxygenated multicarbon products^12,13^, but significant advances remain to be made on optimizing catalyst activity, selectivity, and stability, as well as maximizing the techno-economic merit^14,15^. For that, a profound understanding of the catalytic process dictated by explicit catalyst structure is imperative for realizing product-orientated catalyst design and development^16–18^.

Among the diverse electrocatalysts explored for CO_2_RR, metal-organic frameworks (MOFs) represent a unique category with well-defined and tunable topologic/chemical structure comprising atomically isolated active sites that not only facilitate charge transfer and mass transport, but also help furnish mechanistic understanding on the catalytic process^19,20^. However, most of the reported MOFs, in their pristine form, produce mainly C_1_ products such as CO, HCOOH, and CH_4_^7,21,22^. This probably arises from the distance between separated active sites in the reticular network that prohibit efficient C–C coupling, which otherwise would be more advantageous if the active sites were adjacent to each other in close proximity^23^. As multi-carbon products are often the development target^15^, a key question is how to encourage C–C coupling on the atomically isolated metal sites inside the reticular network?

A further issue of the MOF-based CO_2_RR electrocatalysts lies in their instability in both alkaline and electrochemical conditions^24,25^. Electron transport in the standalone MOFs typically traffics through the node-linker network, which can be demolished under the tough electrolytic condition of CO_2_RR^26,27^. In fact, many previous studies on MOFs as CO_2_RR catalysts have mainly utilized them as the structural precursor or redox mediator after going through post-synthetic treatments for structural mutation and stabilization^24,28,29^. These measures, however, would unavoidably corrupt the well-defined topologic and molecular structure of MOFs, and thus curtail mechanistic understanding^30^. As a result, it is critical to find appropriate techniques to stabilize MOFs while simultaneously boosting redox charge transfer toward selective hydrocarbon synthesis in order to take full advantage of their structural merits^20^.

Impregnation of metal nanostructures into the reticular framework has been demonstrated as an effective way to endow desired functionalities (e.g., conductivity, photoactivity, and catalytic activity) to MOFs^31–33^. When adapted to CO_2_RR, this allows a tandem pathway for producing >2e^−^ products to be established by exploiting different active sites from different structural moieties^34^. In this case, the incorporated metals might effectively alter the charge distribution and conduction path within the MOF framework to further improve the CO_2_RR behavior.

**Fig. 1:**
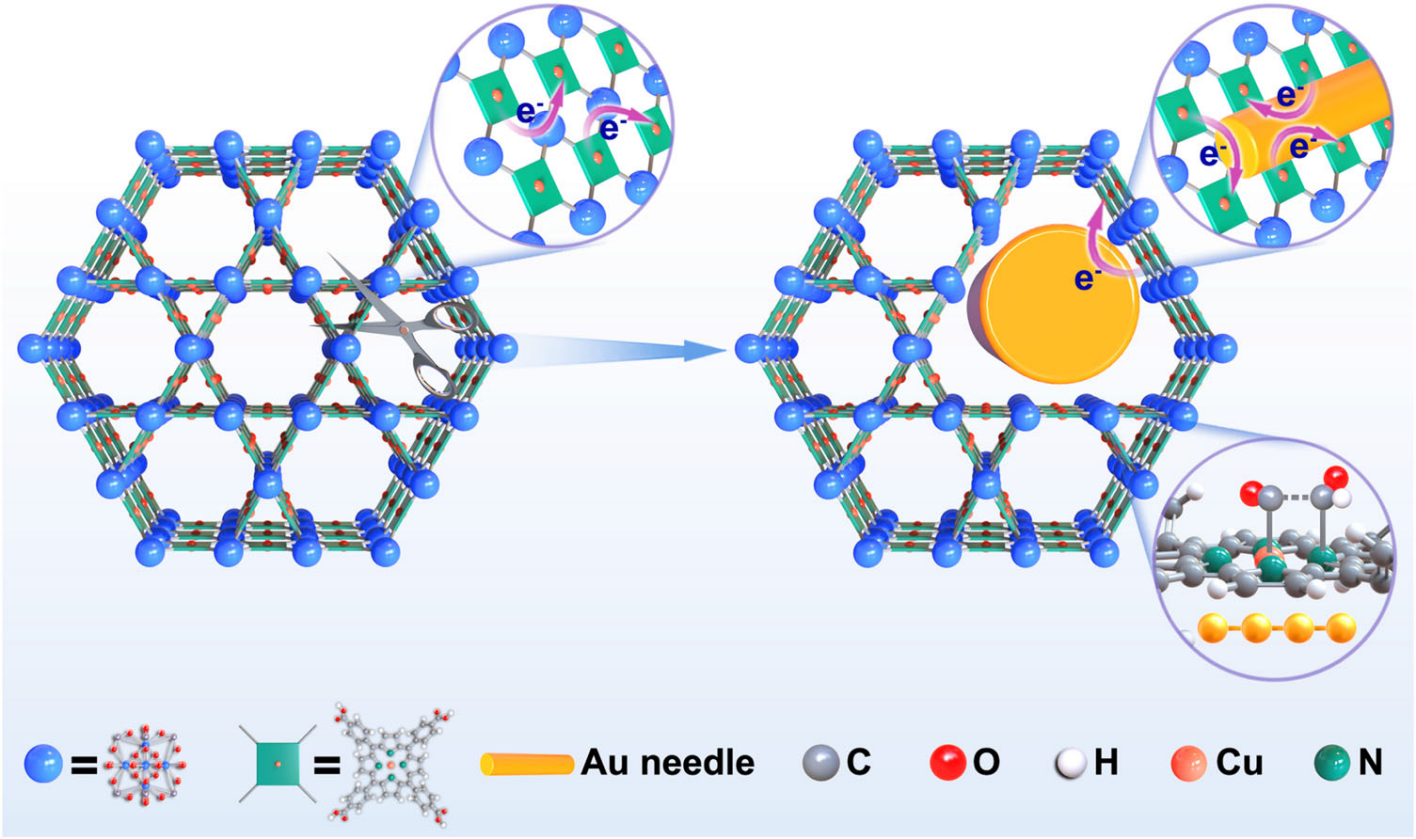
Schematic illustration of the catalytic mechanism. Impregnation of Au nanoneedles into PCN-222(Cu) with cleaved ligand-node linkage to alter the charge conduction path and steer the CO_2_RR pathway.

Herein, we set out to impregnate Au nanoneedles into the Zirconium-based PCN-222 MOF, which has isolated cupric porphyrin centers, to assess its performance as a CO_2_RR catalyst. The idea is to exploit the embedded Au as a high-efficiency CO generator, and the Cu-N_4_ active sites in the metalloporphyrins to relay and convert CO further into >2e^−^ hydrocarbon products with high selectivity (Fig. 1). For control studies, we also synthesize the same PCN-222 MOFs with Au nanoparticles deposited on the surface exterior, as well as MOFs w/o Au and Cu motifs, to interrogate the associated reaction mechanisms. We show that despite the possession of an incoherent framework, the Au-impregnated MOF produces ethylene in a high selectivity that has been rarely seen for such materials. Combining operando synchrotron X-ray spectroscopy, in-situ IR, and DFT modeling, we ascribe the C–C coupling to a tandem mechanism, where CO generated from the impregnated Au nanoneedles are adducted to *CHO at the Au-activated N sites, and the improved structural and catalytic stability to an altered charge conduction path that bypasses the incoherent framework.

## Results

### Catalyst synthesis and characterization

**Fig. 2:**
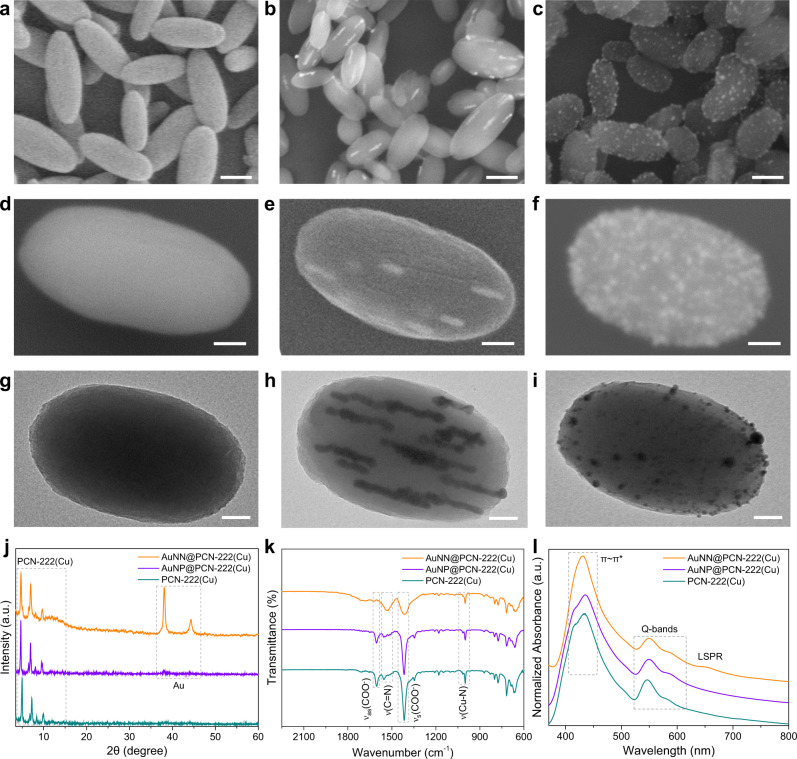
Structural characterizations of the three PCN-222(Cu) catalysts with and w/o Au. **a**–**c** low-magnification, **d–f** high-magnification SEM, and **g**–**i** TEM images of PCN-222(Cu), AuNN@PCN-222(Cu), and AuNP@PCN-222(Cu), respectively. **j**–**l** XRD, FT-IR, and UV-vis spectra of the above three catalysts. The scale bars are 250 nm (**a**–**c**), and 50 nm (**d**–**i**).

The syntheses and characterizations of Zirconium-based PCN-222 (PCN = porous coordination networks) MOFs utilizing tetrakis(4-carboxyphenyl)porphyrin (TCPP) ligands without and with metalloporphyrin Cu centers (designated as PCN-222 and PCN-222(Cu), respectively) are depicted in the Supplementary note 1 and Supplementary Figs. 1–6. Two synthetic protocols were adopted to affix/impregnate Au nanostructures onto/into the PCN-222(Cu) MOF, exhibiting an ellipsoidal morphology (Fig. 2a, d, g). Specifically, when chlorauric acid was reduced by carboxylic groups contained in the MOF ligands at properly adjusted aqueous pH, Au nanoneedles (AuNN) were embedded into the PCN-222(Cu) ellipsoids in alignment with the MOF pore channels. These samples are denoted as AuNN@PCN-222(Cu) (Fig. 2b, e, h). The average diameter of the implanted AuNN is about 10 ± 2 nm, much bigger than that of the larger hexagonal pore channels (3.7 nm) illustrated in Supplementary Fig. 1. Thus, we presume that during the growth of the AuNN, part of the PCN-222(Cu) framework was eroded by cleaving the node-ligand linkage as sketched in Fig. 1. Similar observation was also made by Duan et al^35^ when inserting Au nanowires into MOF-545 in water. In an aqueous environment, the carboxylic groups from the ligand-node redox couple in the MOF can be attacked by the Au^3+^ oxidizer, which in turn cleaves the ligand-node linkage. Without the extra addition of reducing agent, the carboxylates are solely responsible for reducing Au^3+^ to Au^0^. Alternately, when the chlorauric acid was reduced by the extra added NaBH_4_ in ethanol, Au nanoparticles (AuNP) were deposited instantly on the surface of the PCN-222(Cu) ellipsoids (Fig. 2c, f, i), with the resulting products denoted as AuNP@PCN-222(Cu).

Figure 2j compares the XRD patterns of PCN-222(Cu), AuNN@PCN-222(Cu), and AuNP@PCN-222(Cu). We see that the two Au peaks of AuNN@PCN-222(Cu) at 38° and 44° (corresponding to (111) and (200) planes, respectively) are much sharper than those of the AuNP@PCN-222(Cu), indicating that the as-formed Au nanoneedles are better crystalized due to slower growth rate. In contrast, the deposition of Au NPs on PCN-222(Cu) by NaBH_4_ is much faster, resulting in numerous but smaller Au crystallites. At lower 2θ angles, the serial MOF peaks from AuNN@PCN-222(Cu) are not as prominent as those of AuNP@PCN-222(Cu), implying that the reticular framework of AuNN@PCN-222(Cu) is relatively disordered due to the ligand carboxylates reacting with chlorauric acid. This change of coordination network in AuNN@PCN-222(Cu) further leads to a notably different IR spectrum compared to those of AuNP@PCN-222(Cu) and PCN-222(Cu) (Fig. 2k). For AuNN@PCN-222(Cu), both the asymmetric and symmetric stretching of COO^−^ at 1600 and 1416 cm^−1^, respectively, are drastically weakened, whereas the C=N vibration at 1542 cm^−1^ is intensified and redshifted. These could be attributed to the cleavage of the node-linker connection, as well as the structural distortion of the metalloporphyrin ring upon Au impregnation. All samples displayed a strong absorption at 1000 cm^−1^ arising from Cu-N stretching in metalloporphyrins.

The UV-vis spectra of PCN-222(Cu), AuNN@PCN-222(Cu), and AuNP@PCN-222(Cu) all exhibit only two Q bands, affirming the Cu coordination in porphyrin centers (Fig. 2l)^14^. The extra absorption band at 650 nm on AuNN@PCN-222(Cu) can be ascribed to the longitudinal surface plasma resonance (LSPR) of the well-crystalized Au nanoneedles, which is largely governed by the aspect ratio^36^. On the other side, the shoulder of the porphyrin π~π* transition band observed for PCN-222(Cu) and AuNP@PCN-222(Cu) is missing in the spectrum of AuNN@PCN-222(Cu), further confirming a structural distortion of the porphyrin ring^37^, in resonance with the altered C=N vibration observed in IR. Lastly, despite the different reaction protocol and composite structure, elemental analyses by Inductively Coupled Plasma Emission Spectrometry (ICP-ES) revealed similar mass ratios of Au, Cu, and Zr in both AuNN@PCN-222(Cu) and AuNP@PCN-222(Cu) (Supplementary Table 1).

### Electrochemical reduction of CO_2_

The CO_2_RR performances of PCN-222(Cu), AuNN@PCN-222(Cu), and AuNP@PCN-222(Cu) were investigated in an H-cell containing 0.1 M KHCO_3_ saturated with continuously purged CO_2_. Linear scanning voltammograms show that at potentials lower than −0.5 V (vs. RHE, all potentials are referred to this format hereafter) the current density of AuNN@PCN-222(Cu) is slightly lower than that of AuNP@PCN-222(Cu), but higher than that of PCN-222(Cu) (Supplementary Fig. 7), which is further corroborated by electrochemical impedance measurements at −1.2 V, revealing the charge transfer resistance (R_ct_) in the order of AuNP@PCN-222(Cu) < AuNN@PCN-222(Cu) < PCN-222(Cu) (Supplementary Fig. 8). Considering that on AuNP@PCN-222(Cu) the Au nanoparticles are mostly exposed at the surface and the MOF framework is more integral and coherent, the higher charge transfer kinetics could arise from its high electric conductivity combined with its overall catalytic potency (including both HER and CO_2_RR).

**Fig. 3:**
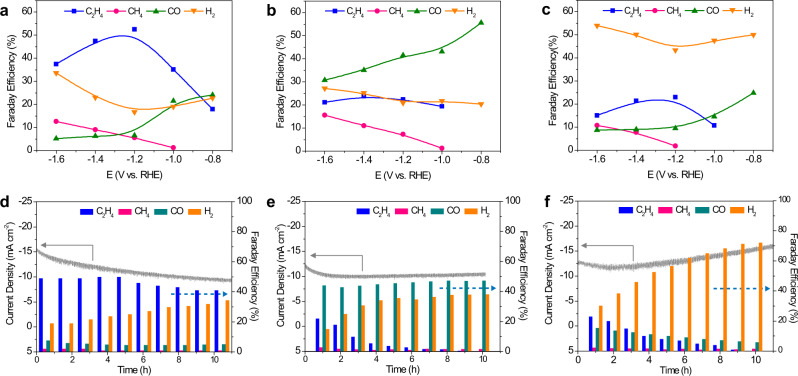
CO_2_RR performances of the catalysts. **a**–**c** FEs of various reduction products for AuNN@PCN-222(Cu), AuNP@PCN-222(Cu), and PCN-222(Cu), respectively, at different potentials. **d**–**f** Chronoamperometric stability tests for AuNN@PCN-222(Cu), AuNP@PCN-222(Cu), and PCN-222(Cu) at −1.2, −1.0, and −1.2 V, respectively, with the gaseous products measured every 1 h.

Potentiostatic measurements by sequentially decreasing the applied potentials from −0.8 to −1.6 V showed that AuNN@PCN-222(Cu) produced mainly C_2_H_4_ with the maximal FE of 52.5% observed at −1.2 V (Fig. 3a). This is, to our best knowledge, among the best seen for MOFs reported in the literature (Supplementary Table 2). For AuNP@PCN-222(Cu), CO constituted the major reduction product over the whole potential range tested, with the FE decreasing with the applied potential from 55.5% at −0.8 V to 30.7% at −1.6 V (Fig. 3b). The high CO yield observed on AuNP@PCN-222(Cu) can be directly related to the surface-exposed Au nanoparticles. In a stark contrast, PCN-222(Cu) produced mainly H_2_ throughout the entire potential range (Fig. 3c). Above all, the FEs of C_2_H_4_ production on AuNN@PCN-222(Cu) are significantly higher than on Au-NP@PCN-222(Cu) and PCN-222(Cu), implying a different mechanism for ethylene formation.

Partial current density plots showed the yield rate of C_2_H_4_ on AuNN@PCN-222(Cu) was nearly three-fold higher than that on AuNP@PCN-222(Cu) and PCN222(Cu) (Supplementary Fig. 9a), indicating that Au nanoneedles inside the MOF help to dramatically boost the kinetics of ethylene production. The difference in CH_4_ production between AuNN@PCN-222(Cu) and AuNP@PCN-222(Cu) was not significant, and both were marginally higher than that on PCN-222(Cu), suggesting that the Au nanostructures exert limited influence on methane production (Supplementary Fig. 9b). On the other hand, the CO production on AuNP@PCN-222(Cu) was almost four times of that on AuNN@PCN-222(Cu) at potentials lower than −1.2 V (Supplementary Fig. 9c), at which C_2_H_4_ reached the highest FE on the latter (Fig. 3a). This suggests that on AuNP@PCN-222(Cu) CO generated by Au nanoparticles are unlikely to re-enter the CO_2_RR pathway to yield >2e^−^ products. We also noted an abnormal drop of CO yield from −1.0 to −1.2 V on AuNN@PCN-222(Cu) (Supplementary Fig. 9c), possibly due to the overwhelming consumption of CO to yield C_2_H_4_ at these potentials. Thus, the production of C_2_H_4_ on AuNN@PCN-222(Cu) presumably follows a tandem path, in which CO generated by AuNN are further reduced on the metalloporphyrin sites. This viewpoint was further reinforced by a series of control experiments with MOFs containing free-base porphyrins and different AuNN loading, as detailed in Supplementary note 2 and Supplementary Figs. 10, 11.

Next, the electrocatalytic stabilities of AuNN@PCN-222(Cu), AuNP@PCN-222(Cu), and PCN-222(Cu) were compared by conducting chronoamperometric i-t tests for a duration of 10 h (Fig. 3d–f). Impressively, AuNN@PCN-222(Cu) manifested a very stable C_2_H_4_ yield during the first 5 h with a nearly constant FE of ~50%. After that, it gradually decreased to 41% at the end of the 10-h test, accompanied with an incremental growth in H_2_ production (Fig. 3d). On AuNP@PCN-222(Cu), the CO production was nearly constant, concomitant with the decrease in C_2_H_4_ yield and increase in H_2_ production (especially in the first 5 h, Fig. 3e). As for PCN-222(Cu), H_2_ production was dominant throughout the test and continually rose at the expense of C_2_H_4_ and CO (Fig. 3f). The varying trend of C_2_H_4_, CO, and H_2_ evolution observed on these catalysts suggest different structural mutation during CO_2_RR, which will be closely inspected by post-mortem and operando studies as detailed below.

### Post-mortem and operando characterizations

**Fig. 4:**
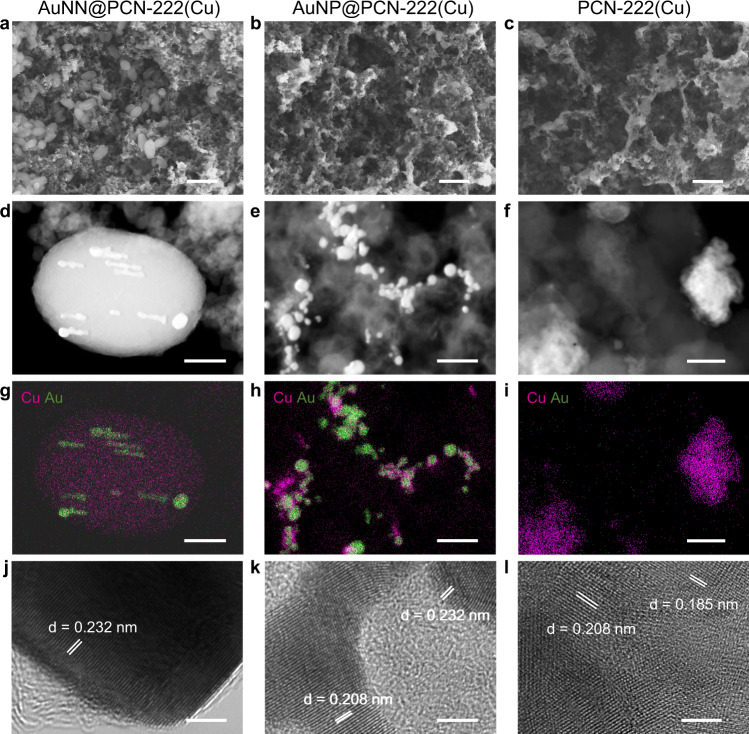
Microstructural characterizations of the three catalysts after 1 h test of CO_2_RR. **a**–**c** SEM, **d**–**f** STEM, **g**–**i** EDX-mapping, **j**–**l** HR-TEM images of AuNN@PCN-222(Cu), AuNP@PCN-222(Cu), and PCN-222(Cu), respectively. The scale bars are 500 nm (**a**–**c**), 100 nm (**d**–**i**), and 3 nm (**j**–**l**).

The catalyst morphologies after one hour of CO_2_RR at −1.2 V were examined using SEM and TEM (Fig. 4). SEM images show while AuNN@PCN-222(Cu) well retained its original ellipsoidal morphology, AuNP@PCN-222(Cu) and PCN-222(Cu) collapsed into irregular shapes (Fig. 4a–c vs. Supplementary Fig. 12), which are better visualized by the high-angle annular dark field-scanning transmission electron microscopy (HAADF-STEM) images shown in Fig. 4d–i. On the post-electrolytic AuNN@PCN-222(Cu) sample, Au nanoneedles impregnated into the PCN-222(Cu) matrix remained mostly intact, and Cu was still homogeneously dispersed (Fig. 4g). By contrast, numerous aggregated metal particles were observed on the post-electrolytic AuNP@PCN-222(Cu) and PCN-222(Cu) samples. EDX-mapping revealed that the nanoparticles evolved from AuNP@PCN-222(Cu) contained both Cu and Au in segregated phases (Fig. 4h). Further analyses using HR-TEM revealed characteristic Au(111) lattice fringes with a d-spacing of 0.232 nm on the Au nanoneedles of post-electrolytic AuNN@PCN-222(Cu) without any Cu-related facets (Fig. 4j). Whereas Cu(111) and Cu(100) facets respectively with d-spacings of 0.208 and 0.185 nm were observed on post-electrolytic PCN-222(Cu) (Fig. 4l), both Au(111) and Cu(111) lattice fringes, as expected, were visualized on the post-electrolytic AuNP@PCN-222(Cu) sample (Fig. 4k).

Moreover, both SEM and TEM images taken on AuNN@PCN-222(Cu) after 10 h of electrolytic CO_2_RR at −1.2 V showed that the integrity of the MOF structure was still mostly retained (Supplementary Fig. 13), despite of a few irregular ellipsoids. This is in great contrast to the completely collapsed morphology of AuNP@PCN222(Cu) and PCN222(Cu) after just 1 h. Overall, the above microscopic observations strongly attest to the structural durability of AuNN@PCN-222(Cu) in comparison to AuNP@PCN-222(Cu) and PCN222(Cu) during CO_2_RR.

**Fig. 5:**
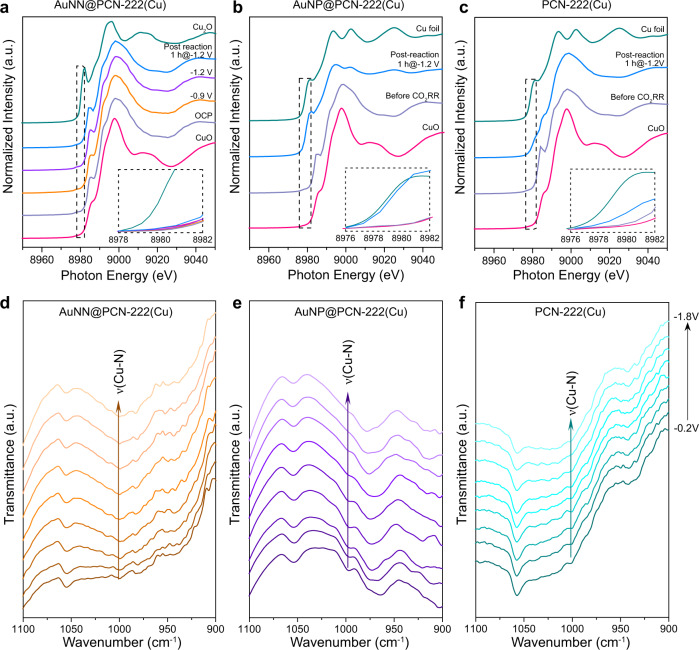
XANES and IR characterizations on the structural stability of catalysts. **a** Operando Cu K-edge XANES spectra taken on AuNN@PCN-222(Cu) at varying potentials and after 1 h CO_2_RR test at −1.2 V. **b,c** Cu K-edge XANES spectra acquired on AuNP@PCN-222(Cu) and PCN-222(Cu) before and after 1 h CO_2_RR test at −1.2 V. **d**−**f** Serial ATR-SEIRAS spectra acquired by ramping down the potential from −0.2 to −1.8 V on AuNN@PCN-222(Cu), AuNP@PCN-222(Cu) and PCN-222(Cu), respectively.

Operando X-ray absorption spectroscopy (XAS) was used to track the evolution of Cu valence and coordination states in AuNN@PCN-222(Cu) during CO_2_RR to further support the above point of view. Previous studies have shown Cu clusters can be reversibly reduced in the Cu-N_4_ complexes prepared pyrolytically^38^ or molecularly^39^, but this is not the case in the current study. In the in-situ X-ray absorption near-edge spectra (XANES) with CuO and Cu_2_O as references, the oxidation state of Cu^2+^ at the metalloporphyrin centers barely changed when the applied potential was ramped from OCP to −1.2 V (Fig. 5a). Furthermore, the XANES spectrum taken on AuNN@PCN-222(Cu) immediately after 10 h of stability test showed only a marginally reduced Cu state close to +2, confirming that the Cu centers were mostly unchanged during the prolonged electrolysis (Supplementary Fig. 14). These results are in good resonance with the structural stability witnessed by microscopies. In comparison, the adsorption edges of post-electrolytic AuNP@PCN-222(Cu) and PCN-222(Cu) downshifted closer to that of the reference Cu foil (Fig. 5b, c), suggesting that Cu^2+^ centers in these samples were reduced, which is further justified by the Cu–Cu bonding in the Fourier-transform extended X-ray absorption fine structure (FT-EXAFS, Supplementary Figs. 15,16). We note that from both the XANES and FT-EXAFS results the metalloporphyrin Cu centers in AuNP@PCN-222(Cu) are more easily to be reduced, likely due to the higher current densities passing through the MOF reticular network.

The structural stability of the catalysts during CO_2_RR was further examined by in-situ attenuated-total-reflection surface-enhanced infrared spectroscopy (ATR-SEIRAS) with the applied potential swept from −0.2 to −1.8 V (Fig. 5d–f). Note that the prominent stretching mode of Cu-N at 1000 cm^−1^ (Fig. 2k) can be used as a good landmark to index the stability of the metalloporphyrin Cu centers. For AuNN@PCN-222(Cu), the Cu-N peak was persistent throughout the entire potential range (Fig. 5d), whereas those of AuNP@PCN-222(Cu) and PCN-222(Cu) decayed gradually with increasing bias. Particularly, at −1.8 V, the Cu-N vibration on AuNP@PCN-222(Cu) was mostly disappeared (Fig. 5e), echoing its liability for reduction as aforementioned. Taken together, both the above XAS and IR spectroscopic observations attest to the better durability of AuNN@PCN-222(Cu) under CO_2_RR conditions, coinciding with its good electrocatalytic stability in C_2_H_4_ production.

**Fig. 6:**
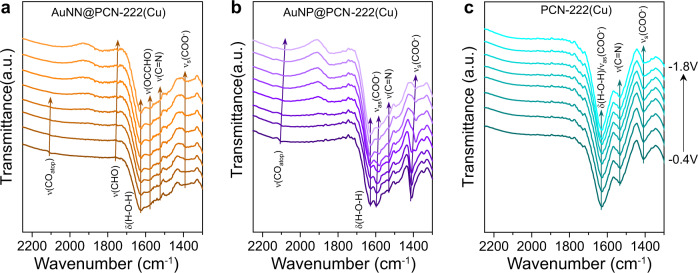
Operando ATR-SEIRAS spectra. **a** AuNN@PCN-222(Cu), **b** AuNP@PCN-222(Cu), and **c** PCN-222(Cu). All spectra were recorded while ramping down the applied potential from −0.4 to −1.8 V to monitor the evolution of intermediates binding.

In-situ ATR-SEIRAS was also exploited to trace the evolution of reaction intermediates during CO_2_RR from −0.4 to −1.8 V (Fig. 6). On AuNN@PCN-222(Cu), the absorption peak at 2108 cm^−1^ can be attributed to the C ≡ O stretching of adsorbed *CO atop Au active sites (CO_atop_)^40^, which gradually decayed and disappeared after −0.8 V, implying the consumption of CO_atop_ at more negative bias for hydrocarbon production (Fig. 6a). This coincides with the CO current plot shown in Supplementary Fig. 9c, where a drop of current density occurred at −1.0 V. The peak at 1750 cm^−1^, intensifying with decreasing potentials, is possibly related to the *CHO absorbed on the metalloporphrins^10^. The *CHO could serve as the precursor for subsequent CH_4_ production, as well as the formation of *CO-CHO by adducting one CO from Au, which is evidenced by the intensifying absorption band at 1578 cm^−1^ 41. The absorption band at 1532 cm^−1^, as aforementioned, is ascribed to the stretching mode of C = N within the porphyrin groups. Collectively, the serial IR spectra acquired on AuNN@PCN-222(Cu) further support the tandem mechanism of C_2_H_4_ formation, where CO generated from AuNN could be adducted to *CHO on the metalloporphyrins, followed by subsequent PCET (proton-coupled electron transfer) processes for hydrogenation^14^.

In the sequential IR spectra of AuNP@PCN-222(Cu), the scenario of intermediates binding is quite different (Fig. 6b). Firstly, the peak of CO_atop_ is more persistent through the applied bias, which is consistent with the observed high CO yield and indicates their consumption to form >2e^−^ products could be less significant. Secondly, the intermediate of *CO-CHO previously observed on AuNN@PCN-222(Cu) with the intensity increasing with decreasing potentials is indistinguishable here. Thirdly, the COO^−^ peaks at 1600 and 1400 cm^−1^ (also prominent in Fig. 2k for AuNP@PCN-222(Cu) and PCN-222(Cu)) dropped their intensity along the reaction course, corroborating the corruption of the MOF structure during CO_2_RR as seen by electron microscopies. As for PCN-222(Cu), the in-situ IR spectra in Fig. 6c display no evident absorptions from the CO_2_RR intermediates due to either the lack of signal enhancement from the localized surface plasmon resonance (LSPR) of Au, or the overall low CO_2_RR performance of the catalyst.

### Mechanistic investigations using density functional theory calculations

It is generally accepted that C_2+_ products are produced through C–C coupling of CO* at two adjacent copper sites^42^. However, the closest distance between adjacent copper sites in PCN-222(Cu) is more than 10 Å (Supplementary Fig. 1), which is too far to enable C–C coupling^21,43–45^. Thus, the C_2_H_4_ production must either be from Cu clusters that were reduced out from the metal-organic frameworks (as evidenced by the above microscopic and spectroscopic observations for AuNP@PCN-222(Cu) and PCN-222(Cu)), or follow a completely different mechanism involving synergy between Au and metalloporphyrins (as suggested by the case of AuNN@PCN-222(Cu)).

We surmise that the decreased C_2_H_4_ production and increased H_2_ yield with time on both AuNP@PCN-222(Cu) and PCN-222(Cu) may be related to maturing of the Cu nanoparticles reduced out from the metalloporphyrins during CO_2_RR, as multi-crystalline Cu crystallites with rich facets and grain boundaries are known to favor C_2+_ production^46–48^.

To help understand the improved C_2+_ selectivity and catalytic stability witnessed on AuNN@PCN-222(Cu), we employed a simplified model of the essential catalyst module by placing the TCPP(Cu) ligand next to a planar Au_12_ cluster (Supplementary Fig. 17). By modulating the distance between the TCPP(Cu) molecule and Au_12_ cluster from 3 to 4.5 Å, we found the lowest energy of the two-body system at 3.5 Å (Supplementary Fig. 17d). Therefore, we used the TCPP(Cu)-Au_12_ model with a distance of 3.5 Å for subsequent calculations and compared it with the standalone TCPP(Cu). Furthermore, we fixed the four carboxylic groups of TCPP in a steady position so as to mimic their presence in the MOF framework. This treatment is important since the ligand in the MOF is relatively constrained when compared to the free molecule.

**Fig. 7:**
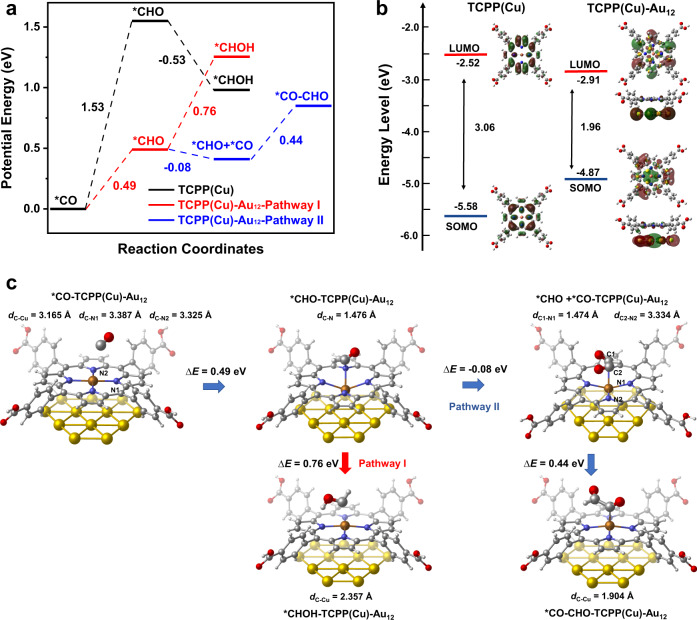
DFT calculations of the configuration and energetics of key intermediates involved in the C1 and C2 paths. **a** The formation energy of key intermediates along with the reaction coordinates for TCPP(Cu) and TCPP(Cu)-Au_12_. **b** Calculated energy levels of HOMO and LUMO for TCPP(Cu) and TCPP(Cu)-Au_12_. **c** Configuration of intermediates absorption on TCPP(Cu)-Au_12_. The colors are C in silver, H in white, O in red, N in blue, Au in yellow, and Cu in orange.

We then performed DFT calculations on the configuration and energetics of key intermediates involved in the C1 and C2 routes. For C1, we considered *CO → *CHO → *CHOH. For C2, we considered *CO → *CHO → *CO-CHO^42,49^. On TCPP(Cu), the formation energy of *CO → *CHO is 1.53 eV (Fig. 7a). After geometry optimization, both *CO and *CHO adsorb on top of the Cu site through C-terminus (with the C-Cu distances of 3.030 and 1.927 Å, respectively), imposing negligible impact on the TCPP(Cu) ring structure that keeps planar (Supplementary Fig. 18). Reducing *CHO to *CHOH is energetically favorable by −0.53 eV. On TCPP(Cu), because each TCPP(Cu) module can only provide one catalytic center for CO_2_ activation at a time, the C–C coupling is unfeasible.

The scenario on TCPP(Cu)-Au_12_ is quite different, where C–C coupling becomes possible (Fig. 7a). Beyond *CHO, both the aforementioned C1 and C2 paths are feasible, while competing with each other. On TCPP(Cu)-Au_12_, the formation energy of *CHO from *CO is 0.49 eV, significantly lower than 1.53 eV on TCPP(Cu). Mullikan Charge analysis shows that the Au_12_ substrate formally donates 0.038 electrons to Cu (Table S4), which weakens the Cu-N binding and enhances the adsorption of both *CO and *CHO (Table S5) that facilitates subsequent reaction cascade.

Different from the case of TCPP(Cu), the adsorbed CO slightly deviates from the top of the Cu site, interacting with the two nearby N atoms (as shown in Fig. 7c with the C-Cu, C-N1, and C-N2 distances of 3.165, 3.387, and 3.325 Å, respectively). In the next step, *CHO moves to the N site rather than residing on the Cu site, forming a C–N bond with a distance of 1.476 Å (Fig. 7c, middle panel). Simultaneously, the Cu-N_4_ motif undergoes an out-of-plane displacement toward the Au plane, freeing the Cu center to accept another Au-generated CO in affording the co-adsorption of *CO and *CHO with an exothermic energy difference of −0.08 eV. Subsequently, C–C coupling occurs by bridging the two intermediates to form *CO-CHO with a surmountable energy of 0.44 eV (Fig. 7c Pathway II). The existence of *CO-CHO intermediate has been confirmed by operando IR at 1578 cm^−1^, which also explains the fast consumption of CO_atop_ on Au at 2108 cm^−1^. Energetically, the C1 route via *CHOH cannot compete with C–C coupling due to its high formation energy of 0.76 eV (Fig. 7c Pathway I).

The N-site catalyzed *CO reduction to *CHO was unexpected, because usually only the metal center of a macrocycle molecule is responsible for the catalytic activity. Here the weakened Cu-N interaction and the transition from D_4h_ to C_4v_ symmetry at the metal center due to AuNN insertion and intermediates adsorption is postulated as the underlying reason for the emerging catalytic activity of the N site. On top of that, AuNN introduced into the MOF interior would further increase the concentration of CO trapped inside the MOF tunnels, due to the spatial confinement effect^50,51^. Such increase in CO concentration would not only improve the probability of C–C coupling when the active sites are brought to sufficient proximity, but also boost the CO_2_RR kinetics^52^.

To testify the role of the MOF framework in promoting C–C coupling, we further conducted control experiments by loading TCPP(Cu) molecules onto Au nanorods/nanoparticles that are lack of spatial confinement and compared their CO_2_RR performance with that of AuNN-PCN-222(Cu) under the same electrolytic condition (Supplementary Fig. 19). In both cases, apart from a small amount of C_2_H_4_, CO and H_2_ constituted the main reduction products, implying the Au nanostructures and TCPP(Cu) functioned separately. Collectively, both the experimental observations and DFT calculations corroborate that introducing AuNN into the framework of PCN-222(Cu) enables to boost C_2_ production.

Ligand-to-metal charge transfer (LMCT) that readily reduces the Cu site in organometallic compounds has been often perceived as the cause of MOF destruction during CO_2_RR^48,49^. Such a process is plausible because the electronic structure of single occupied molecular orbital (SOMO) and lowest unoccupied molecular orbital (LUMO) indicates that the Cu^2+^ can be easily reduced. Therefore, in AuNP@PCN-222(Cu) and PCN-222 (Cu) the centric Cu atom can be readily reduced and dissociated from the porphyrin ring, forming clusters and particles. By contrast, with Au support the frontier orbitals of TCPP(Cu)-Au_12_ move to Au, allowing the charge conductance to bypass the LMCT process, but directly hop from Au to the metalloporphyrin center to attend the PCET process. This is especially for the case of AuNN@PCN-222(Cu) where the MOF skeleton is partially cleaved and the conducting pathway along the node-linker network is broken. Such electron conductance ability of Au provides extra protection of the catalyst center, which explains the significantly extended stability as observed experimentally.

## Discussion

In this study, Zirconium-based PCN-222 MOFs comprising metalloporphyrin Cu centers and impregnated Au nanoneedles were successfully synthesized by exploiting the ligand carboxylates as the reducing agent. Compared to the same MOF structure with surface-deposited Au nanoparticles and the one without Au moieties, AuNN@PCN-222(Cu) demonstrated significantly improved ethylene production up to an FE of 52.5%, whereas AuNP@PCN-222(Cu) and PCN-222(Cu) produced mainly CO and H_2_, respectively. More impressive, AuNN@PCN-222(Cu) manifested better structural stability during CO_2_RR, despite the incoherent reticular network.

Through extensive post-electrolytic and operando characterizations, in conjunction with DFT modeling, the enhanced C–C coupling on AuNN@PCN-222(Cu) was ascribed to a tandem mechanism, where CO generated from the impregnated Au nanoneedles are adducted to *CHO on the metalloporphyrins with Au-activated N motifs. Additionally, its enhanced structural stability during CO_2_RR can be attributed to an altered charge conduction path bypassing the MOF reticular network. By impregnating metal nanostructures into the PCN framework and activating the metalloporphyrin centers, this study sheds new light on boosting the C_2+_ selectivity and catalytic stability by exquisite catalyst design and synthesis.

## Methods

### Synthesis of PCN-222(M)

A traditional solvothermal reaction method was used to synthesize PCN-222(M) (M = H or Cu). ZrOCl_2_·8H_2_O (30 mg), M-TCPP (tetrakis(4-carboxyphenyl)porphyrin, M = H or Cu, 10 mg), and benzoic acid (280 mg) in a mixed solution of DMF (N,N-dimethyl formamide, 10 mL) and DI-water (100 μL) were ultrasonically dissolved in a 20 mL Pyrex vial. The mixture was heated at 90 °C for 8 h in an oil bath with stirring kept during the whole reaction. Afterward, the mixture was cooled down in the water and then centrifuged four times with DI-water and ethanol (8600 g, 15 min). Finally, the product was dissolved into acetone for a week to remove any guest molecules in pores. The dark purple powder obtained after centrifugation and drying were then collected for further use.

### Synthesis of AuNN@PCN-222(Cu)

10 mg of the above-synthesized PCN-222(Cu) was ultrasonically dissolved into 100 mL DI-water buffered with 10 mL 0.1 M KHCO_3_ for controlling the pH (solution A). Then, 100 μL of the HAuCl_4_ aqueous solution (0.05 mol L^−1^) was diluted into 50 mL DI-water and slowly added to solution A at a rate of one milliliter per minute. Under the mild acidic condition, Au^3+^ was reduced by carboxylates contained in the MOF ligands and crystallized into nanoneedles. The amount of Au nanoneedles can be adjusted by varying the added volume of HAuCl_4_. The final product was collected upon centrifugation and drying.

### Synthesis of AuNP@PCN-222(Cu)

10 mg PCN-222(Cu) was ultrasonically dissolved in 100 mL ethanol with the addition of 100 μL HAuCl_4_ aqueous solution (0.05 mol L^−1^). After stirring for 30 min, 0.5 mL NaBH_4_ ethanol solution (50 mmol L^−1^) was added to reduce Au^3+^ into nanoparticles deposited on the MOF surface quickly. After continuously stirring for another ten minutes, the product was collected by centrifugation, followed by freeze-drying overnight.

### Electrochemical measurements

The electrochemical CO_2_RR experiments were carried out in a traditional airtight H-cell controlled with an electrochemical workstation (CHI760E). The H-cell used glassy carbon as a working electrode separated from a Pt counter electrode by a Nafion 115 membrane. The reaction was carried out in a CO_2_-saturated aqueous solution of 0.1 M KHCO_3_. 4 mg catalyst, and 2 mg ketjen black were ultrasonically dissolved in 1 ml ethanol with 50 μL nafion to prepare the catalyst ink. The working electrodes were prepared by drop-casting the catalyst ink on the glassy carbon electrode with a coating area of ~0.2 cm^2^. High-purity CO_2_ gas of 20 cm^3^ min^−1^ was supplied to the gas chamber controlled by a digital mass flow controller (Horiba). The gas products were quantitatively analyzed using gas chromatography equipped with both flame ionization and thermal conductivity detectors (Agilent 7890B). All the potentials were converted to RHE, according to the equation E (vs RHE) = E (vs Ag/AgCl) + 0.059 pH + 0.198.

### Operando ATR-SEIRAS measurements

The ATR-SEIRAS measurements were conducted in a two-compartment spectro-electrochemical cell comprising three electrodes, including a Si prism deposited with a layer of 60 nm Au film as the working electrode, a platinum-wire as the counter electrode, and a standard Ag/AgCl electrode as the reference, as is shown in supplementary Fig. 20. The catalyst ink was spray-coated on the working electrode with an electric airbrush. All electrochemical tests were tested in a 0.1 M KHCO_3_ aqueous solution saturated with a constant CO_2_ flow and controlled by a CHI electrochemical workstation (CHI760E). The cathodic potential was swept from −0.4 V to −1.8 V vs. RHE every 200 mV. All the ATR-SEIRAS spectra were acquired using a Fourier Transform Infrared Spectrophotometer (FT-IR, Nicolet iS50, Thermo Fischer scientific.) equipped with a mercury cadmium telluride (MCT) detector. A bare Au film without loading any catalysts was used as the background reference. All spectroscopic measurements performed at a spectral resolution of 4 cm^−1^ and overlaying of 64 repetitions.

### Operando XAS Measurements

Operando XAS spectra of Cu K-edge were acquired at the Shanghai Synchrotron Radiation Facility, Beamline BL11B beamline. All spectra were recorded in the fluorescence mode using a Lytle detector with the energy calibrated using a Cu foil. The illumination area on the sample was about 200 × 250 μm^2^ (H × V). The electron storage ring was operated at 3.5 GeV in the top-up mode with a beam current of 220 mA. A Si(111) double-crystal monochromator with the energy resolution of ΔE/E = ~1.4 × 10^−4^ was used to monochromatize the incident photons. A pair of Rh-coated mirrors at 4 mrad was employed to reject higher harmonics. A two-compartment H cell was utilized to conduct the electrolysis in CO_2_-saturated 0.1 M KHCO_3_ solution, with the sample set at 45° related to the incident beam. The preparation of electrodes followed the same procedure described in the section of electrochemical measurement. During the measurements CO_2_ was constantly bubbled into the cell at a fixed flow rate. All spectra were acquired under the potentiostatic mode by sweeping the potential from 0 to −1.2 V^53^. Specifically, at −1.2 V, the current density on AuNN@PCN-222(Cu) was ~10 mA cm^−2^, similar to that of the H-cell setup for performance evaluation.

### DFT calculations

DFT calculations were performed using the Gaussian 16 program (version A. 03)^54^ at the level of PBE0 functional^55^ with D3 dispersion correction^56^. Cu and Au were described by the Stuttgart–Dresden pseudopotential and double-ξ valence (SDD) basis set, while H, C, N, O were described by the 6-31 G(d) all-electron basis set. The solvation effect was considered with the continuum solvent model (SMD) with the parameters for water^57^. The time-dependent density functional theory^58,59^ (TD-DFT) was used to predict the frontier molecular orbitals of the Cu-porphyrin w/o Au_12_ cluster. To investigate the CO_2_RR performance of TCPP(Cu) and TCPP(Cu)-Au_12_ systems, the catalysis center of TCPP(Cu) was modeled by cutting the Cu porphyrin structure from single-crystal X-ray diffraction MOF data^44^. The breaking bonds were saturated as benzoic acids to keep the system electrically neutral. Within the available computational cost, the Au_12_ cluster model was employed to represent gold nanoneedles because its work function is close to bulk Au, and the interatomic distance of Au atoms in the Au_12_ cluster is also comparable to that of bulk Au. No significant change of Au_12_ planar structure was observed in the calculation, legitimizing its representation of the rigid Au nanoneedles.

## Supplementary information

Supplementary Info

## Data Availability

All data generated or analysed during this study are included in this published article (and its Supplementary Information files) or can be obtained from the authors on reasonable request.
