# Supplementary material for: Au-activated N motifs in non-coherent cupric porphyrin metal organic frameworks for promoting and stabilizing ethylene production
Source: Nat Commun. 2022 Jan 17;13:63. doi: 10.1038/s41467-021-27768-6 (PMC8763919; doi:10.1038/s41467-021-27768-6)
Supplement: Supplementary file 1 — Supplementary Info [file 41467_2021_27768_MOESM1_ESM.pdf]

Supporting Information

Au-activated N motifs in Non-coherent Cupric Porphyrin Metal Organic Frameworks for Promoting and Stabilizing Ethylene Production

Xulan Xie[1,2,†], Xiang Zhang[1,2,†], Miao Xie[3,†], Likun Xiong[4], Hao Sun[1,2,5], Yongtao Lu[1,2], Qiaoqiao Mu[1,2], Mark H Rummeli[1,2], Jiabin Xu[3], Shuo Li[3], Jun Zhong[3], Zhao Deng[1,2], Bingyun Ma[3], Tao Cheng[3], William A Goddard III[6]*, Yang Peng[1,2,5]**

[1] Soochow Institute for Energy and Materials Innovations, College of Energy, Soochow University, Suzhou 215006, P. R. China.

[2] Key Laboratory of Advanced Carbon Materials and Wearable Energy Technologies of Jiangsu Province, Suzhou 215006, P. R. China.

[3] Institute of Functional Nano and Soft Materials (FUNSOM), Soochow University, Suzhou 215123, China

[4] School of Chemical and Environmental Engineering, Shanghai Institute of Technology, Shanghai 201418, China.

[5] Jiangsu Engineering Laboratory of New Materials for Sewage Treatment and Recycling, Suzhou, 215123, China

[6] Materials and Process Simulation Center, Department of Chemistry, California Institute of Technology, Pasadena, California 91125, United States

[†] contributed equally to this work.

* Corresponding author. E-mail: tcheng@suda.edu.cn; wag@caltech.edu; ypeng@suda.edu.cn

Supplementary Methods

Chemicals

Zirconium dichloride oxide octahydrate ($ZrOCl_2 \cdot 8H_2O$, 98.0%), N,N-Dimethylformamide (C_3H_7ON, 99.9%), and potassium bicarbonate ($KHCO_3$, ≥99.5%) were purchased from J&K Scientific Co., Ltd.. Gold(Ⅲ) chloride hydrate ($HAuCl_4 \cdot xH_2O$, 49-51% Au), Benzoic acid ($C_7H_6O_2$, 99.5%), and sodium borohydride ($NaBH_4$, 98%) were purchased from Shanghai Aladdin Bio-Chem Technology Co., Ltd.. Tetrakis(4-carboxyphenyl)porphyrin (TCPP, $C_{48}H_{30}N_4O_8$, 97%) was purchased from shanghai Kaiyulin Pharmaceutical Co., Ltd..

Characterizations

The crystalline structure of all samples was inspected by X-ray diffraction (XRD, Bruker AXS D8 Advance diffractometer with Cu Kα source). The chemical structure was examined by Fourier-transform infrared spectroscopy (FTIR, Thermo Fisher Scientific) and UV-visible spectroscopy (UV-is, PerkinElmer). The Brunauer-Emmett-Teller surface area of all samples was quantified by N_2 adsorption-desorption isotherm (BET, Tristar II 3020). The surface morphology and micro-structure of all samples were observed using a dual-beam electron microscope (SEM, FEI Scios) and spherical-aberration-corrected TEM (Cs-TEM, FEI Titan Themis Cubed G2 300). Inductively coupled plasma optical emission spectroscopy (ICP-OES, PerkinElmer Optima 8000) was employed to analyze the elemental composition quantitatively. X-ray Absorption Near-Edge Structure (XANES) and Extended X-ray Absorption Fine Structure (EXAFS) data were collected at Beamline 14W from the Shanghai Synchrotron Radiation Facility (SSRF).

Supplementary Figures and Notes

Supplementary Note 1 - Characterizations of PCN-222 and PCN-222(Cu)

The syntheses of Zirconium-based PCN-222 (PCN = porous coordination networks, Supplementary Fig. 1) without and with Cu metalloporphyrin centers (designated as PCN-222(Cu)) utilizing a solvothermal method are detailed in the experimental section. The as-obtained powder of PCN-222 comprising the free-base TCPP (tetrakis(4-carboxyphenyl)porphyrin) ligands exhibits a dark brown color, whereas PCN-222(Cu) is brick red (Supplementary Fig. 2a, b). Both PCN-222 and PCN-222(Cu) show the same ellipsoidal morphology with an average length of 380 ± 17 nm (Fig. 2, Supplementary Fig. 2c, d). X-ray diffraction (XRD) patterns reveal no shift of 2θ angles for the two samples, both matching the documented CCDC No. 893545 for PCN-222 (Supplementary Fig. 3). Fourier transform infrared spectrum (FTIR) of PCN-222(Cu) shows a prominent absorption at 1001 (s) cm^{-1} ascribed to the Cu-N coordination, which is absent in the spectrum of PCN-222 (Supplementary Fig. 4). Furthermore, UV-vis measurements revealed that upon Cu coordination the four Q bands of the free-base porphyrin turned into two in both the PCN-222(Cu) and TCPP(Cu) samples, owing to the degenerated π orbital energy levels as a result of the improved symmetry in metalloporphyrins (Supplementary Fig. 5). N_2 adsorption-desorption isotherms indicate the adoption of metalloporphyrins does not affect the pore characteristics of the MOF, and the measured Brunauer-Emmett-Teller (BET) surface areas are 2406 and 2404 $cm^2\ g^{-1}$ for PCN-222 and PCN-222(Cu), respectively (Supplementary Fig. 6). Note that the type-IV(b) isotherms with two steep rises in N_2 uptake indicate the existence of two types of micro-pores within the MOFs, coinciding with the topological structure shown in Supplementary Fig. 1.

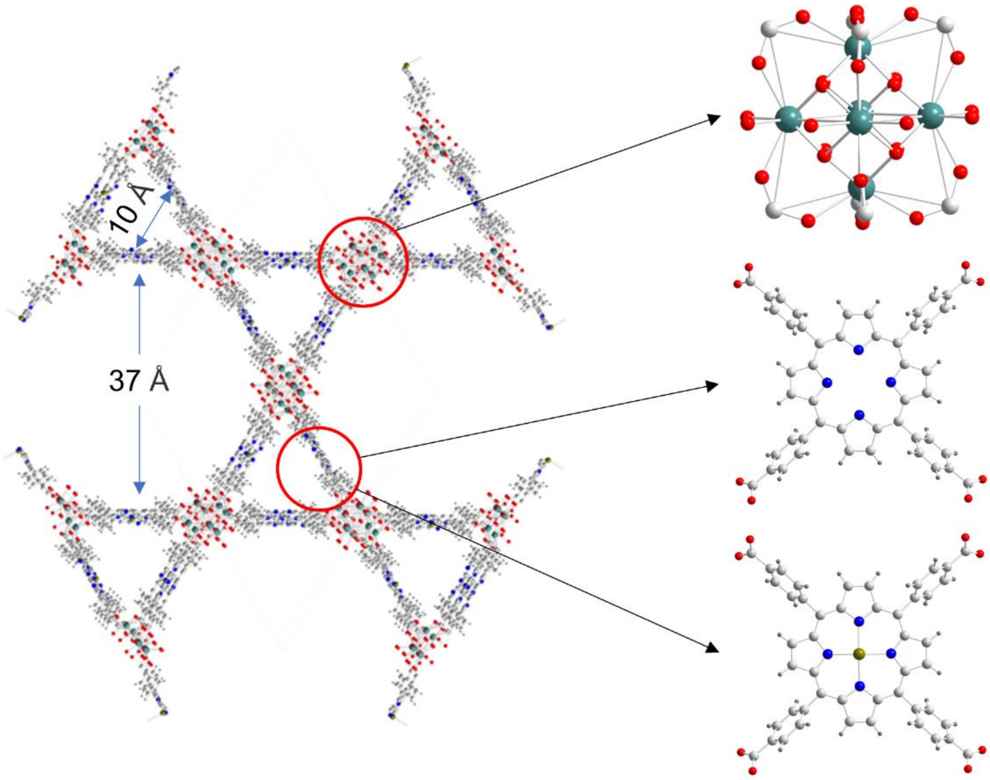

Supplementary Fig. 1. Crystal structure of PCN-222 and PCN-222(Cu).

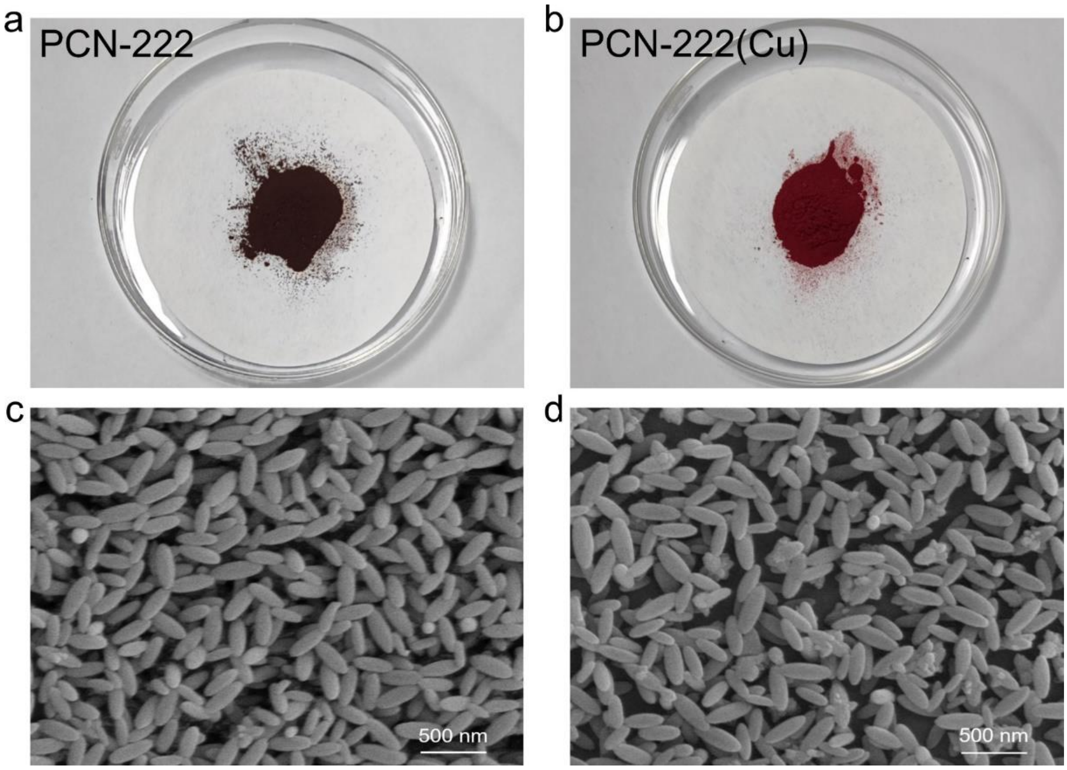

Supplementary Fig. 2. (a, b) Optical and (c, d) SEM images of PCN-222 and PCN-222(Cu), respectively.

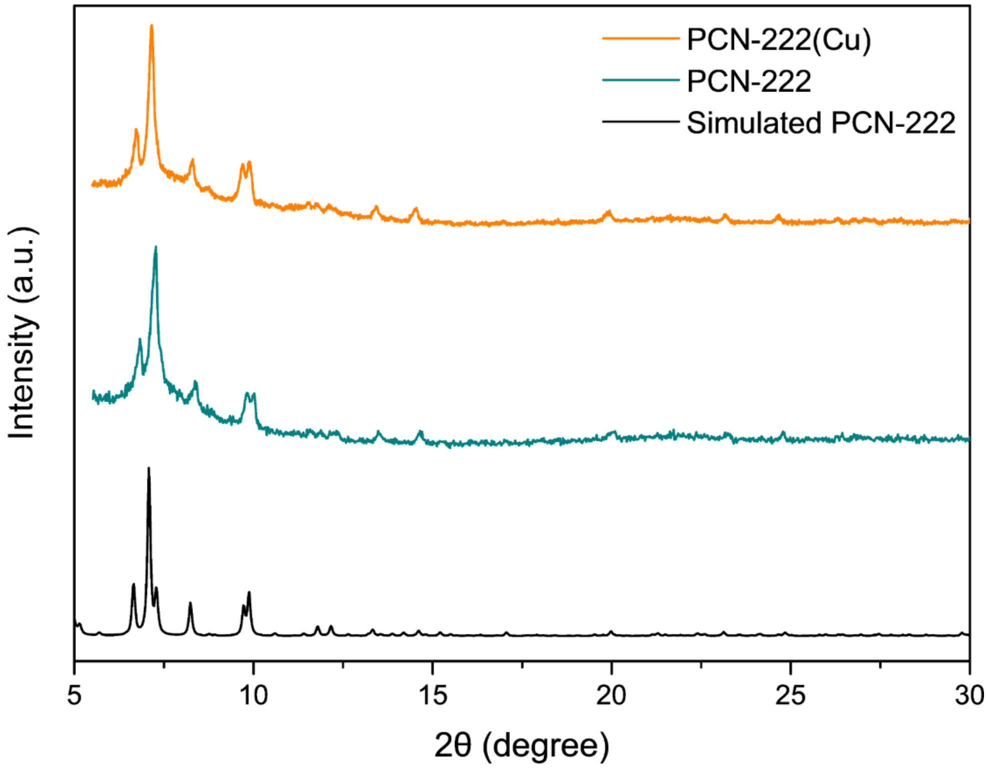

Supplementary Fig. 3. XRD patterns of PCN-222 and PCN-222(Cu).

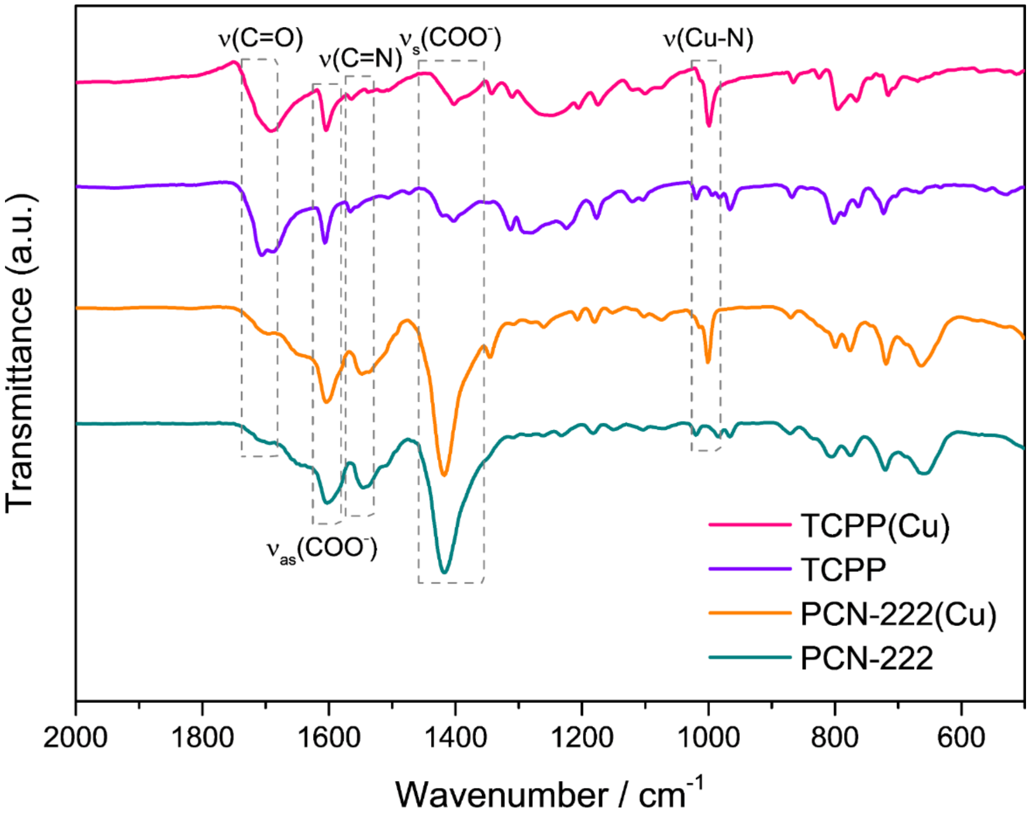

Supplementary Fig. 4. FTIR spectra of PCN-222 and PCN-222(Cu) in comparison with TCPP and TCPP(Cu).

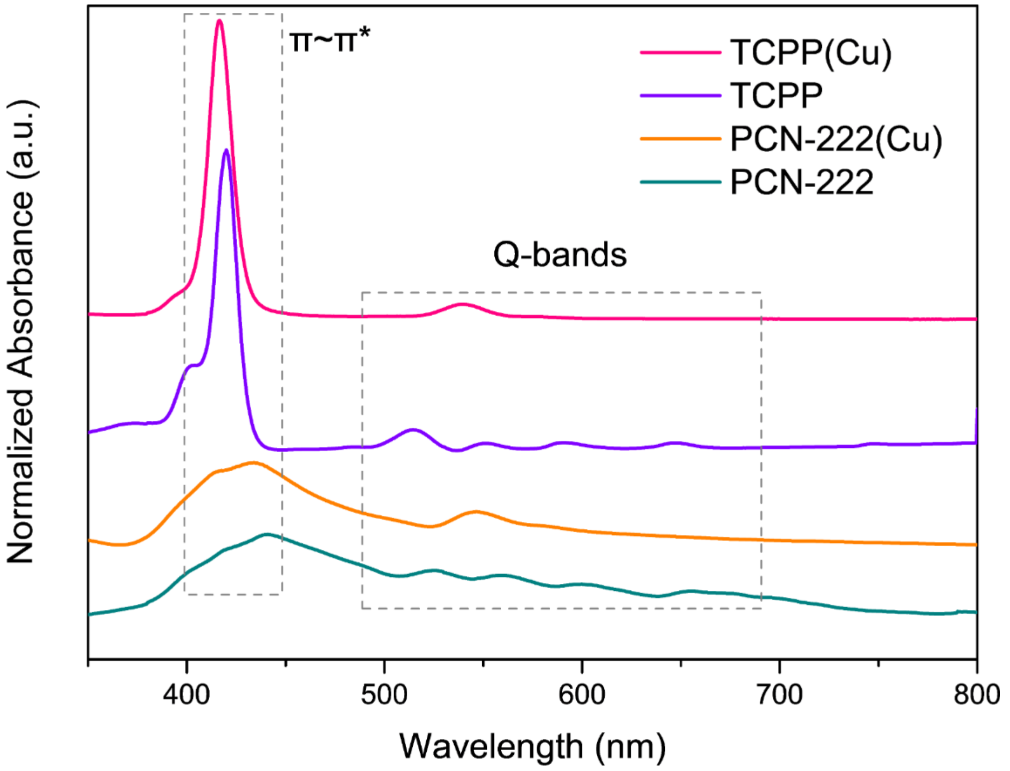

Supplementary Fig. 5. UV-vis spectra of PCN-222 and PCN-222(Cu) in comparison with TCPP and TCPP(Cu).

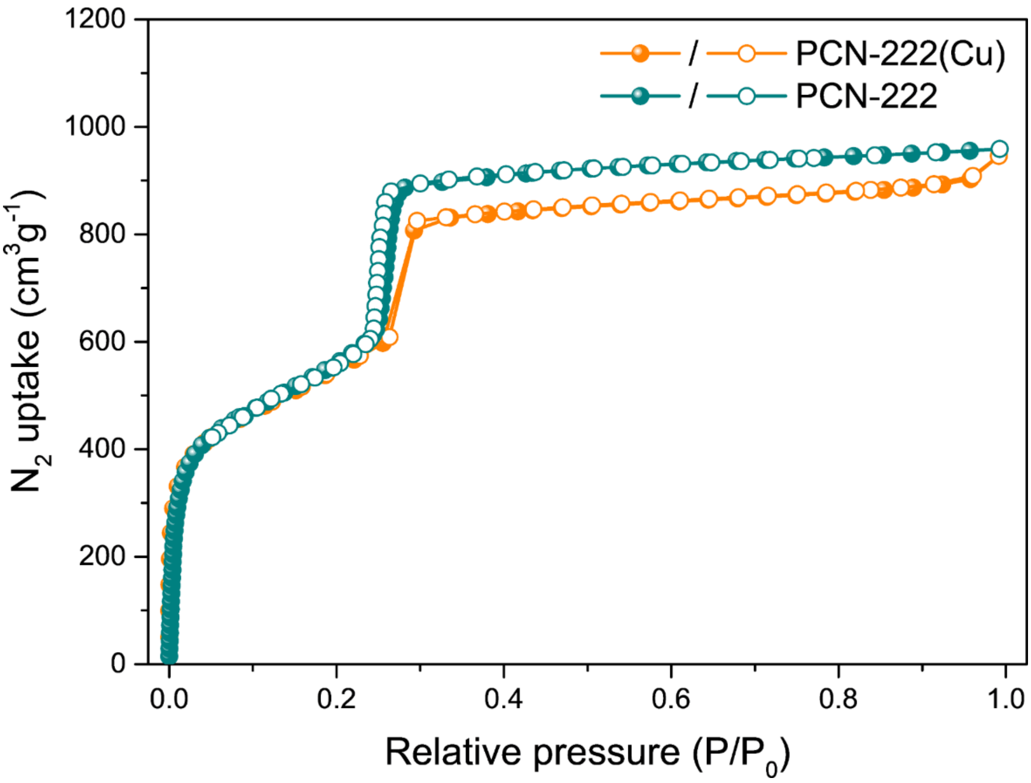

Supplementary Fig. 6. N_2 adsorption-desorption isotherms of PCN-222 and PCN-222(Cu) at 77K.

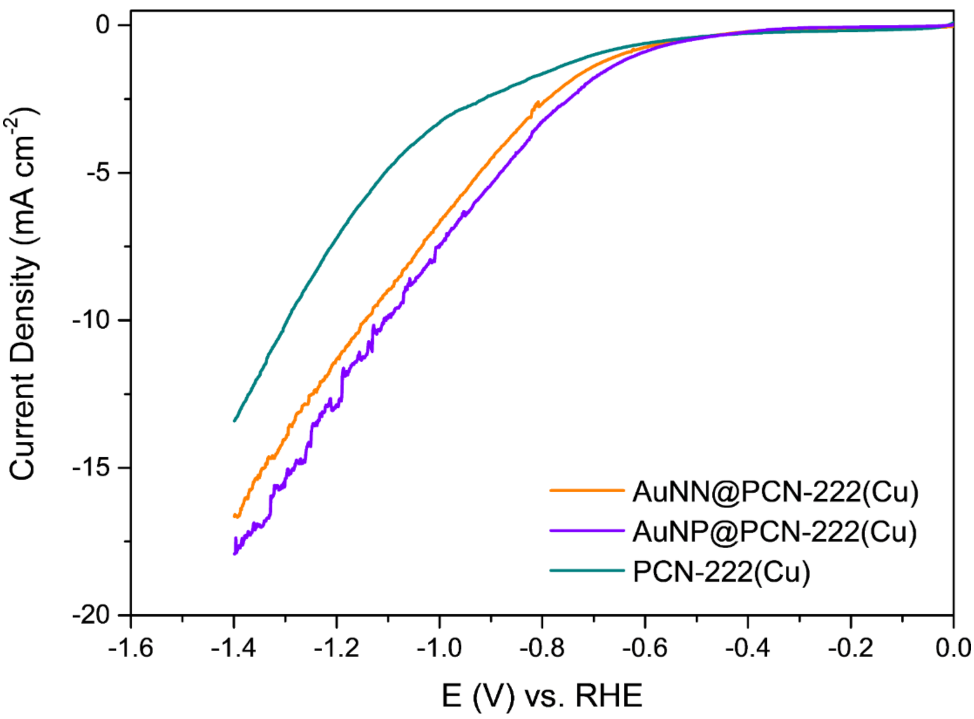

Supplementary Fig. 7. Linear scanning voltammetries of CO_2RR for PCN-222(Cu), AuNN@PCN-222(Cu), and AuNP@PCN-222(Cu).

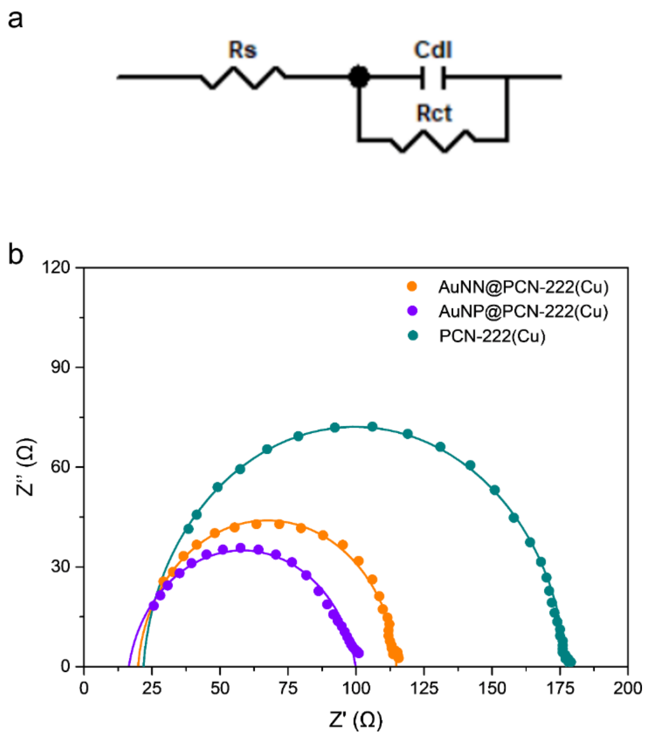

Supplementary Fig. 8. (a) Randles' equivalent circuit used for fitting the experimental impedance data. (b) Nyquist plots acquired at -1.2 V (vs. RHE) for PCN-222(Cu), AuNN@PCN-222(Cu), and AuNP@PCN-222(Cu).

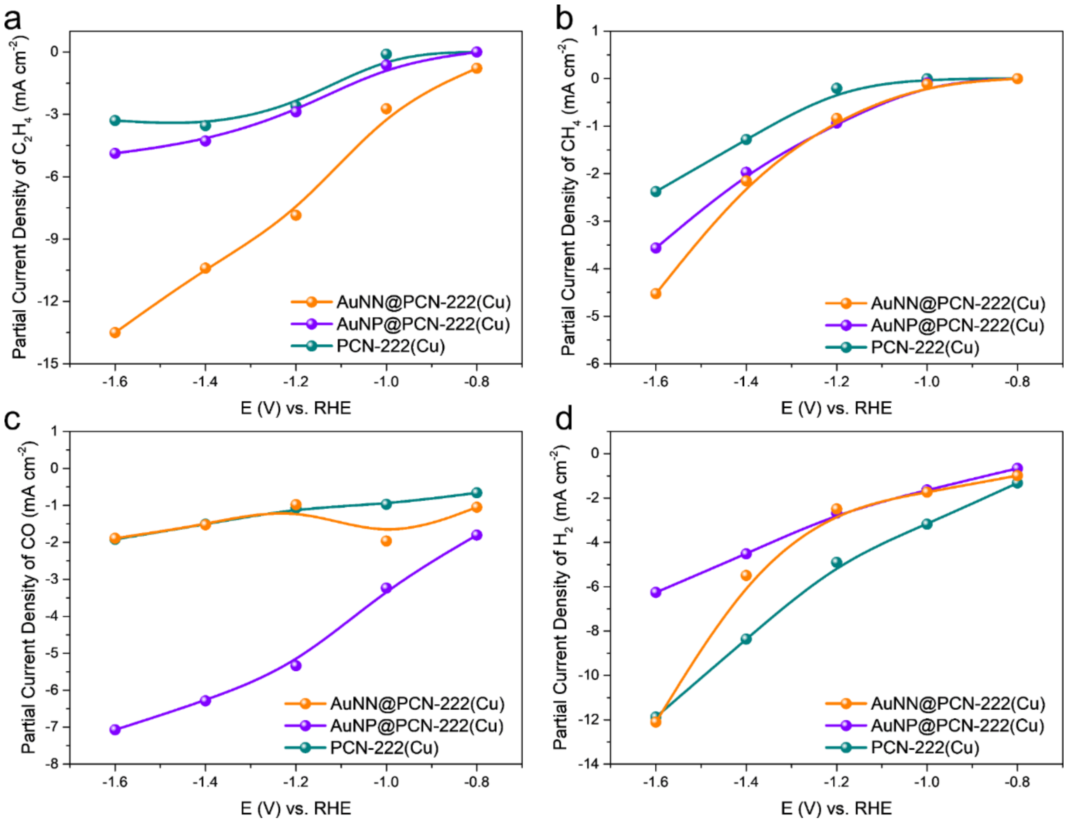

Supplementary Fig. 9. Partial current density plots of different reduction products at varying potentials for PCN-222(Cu), AuNN@PCN-222(Cu), and AuNP@PCN-222(Cu). (a) C_2H_4. (b) CH_4. (c) CO. (d) H_2.

Supplementary Note 2 - Control experiments to verify the tandem pathway for producing C_2H_4 on AuNN@PCN-222(Cu)

For control studies, we also fabricated a group of contrast samples without the metalloporphyrin Cu centers, namely PCN-222, AuNN@PCN-222, AuNP@PCN-222, as well as an AuNN(L)@PCN-222(Cu) sample with less Au nanoneedles impregnated (Supplementary Fig. 10). Without both Au and Cu moieties, PCN-222 produced H_2 as the dominant reduction product with nearly 100% FE in the whole potential range (Supplementary Fig. 11a). By contrast, both AuNN@PCN-222 and AuNP@PCN-222 yielded majorly CO, a small fraction of H_2, but no CH_4 and C_2H_4 (Supplementary Fig. 11b, c). The H_2 generation on AuNN@PCN-222 is slightly higher than that on AuNP@PCN-222, coinciding with the H_2 current density plots in Supplementary Fig. 9d. Compared to AuNN@PCN-222(Cu), AuNN(L)@PCN-222(Cu) yielded less C_2H_4 and CO, but more H_2 (Supplementary Fig. 11d). Collectively from the above observations, it is obvious that the metalloporphyrin Cu centers are indispensable for producing >$2e^-$ products, and a tandem mechanism is likely for AuNN@PCN-222(Cu) to convert CO produced by Au into C_{2+} products on the metalloporphyrins.

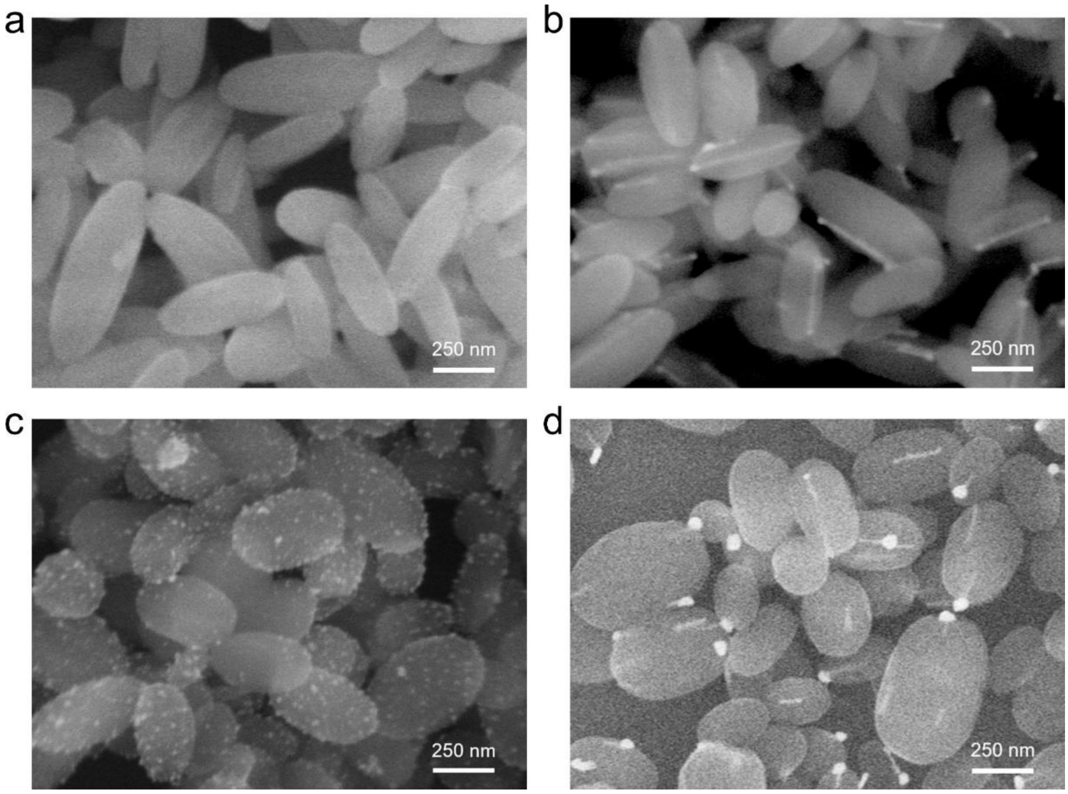

Supplementary Fig. 10. SEM images of (a) PCN-222, (b) AuNN@PCN-222, (c) AuNP@PCN-222 and (d) AuNN(L)@PCN-222(Cu).

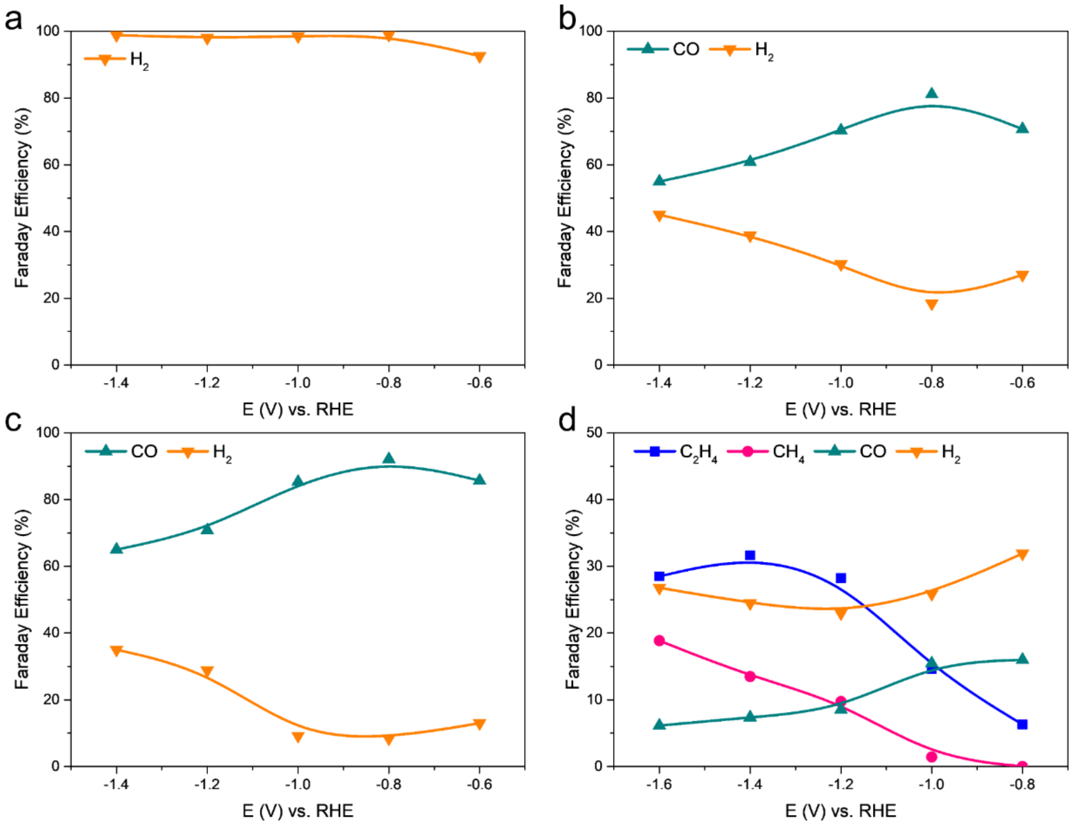

Supplementary Fig. 11. FEs of different reduction products for (a) PCN-222, (b) AuNN@PCN-222, (c) AuNP@PCN-222 and (d) AuNN(L)@PCN-222(Cu) at varying potentials.

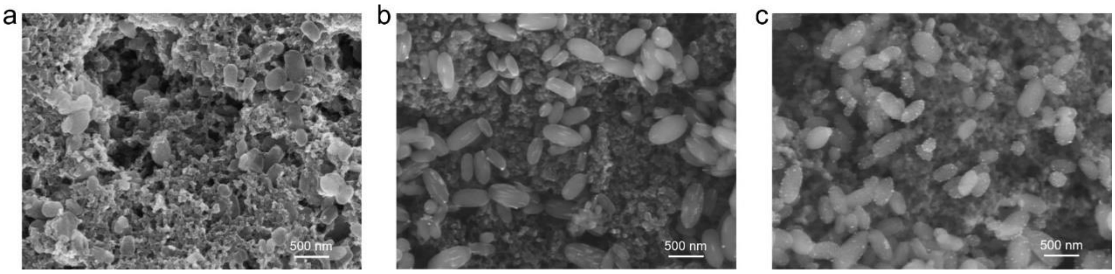

Supplementary Fig. 12. SEM images of the pre-electrolytic catalysts on GDE. (a) AuNN@PCN-222(Cu). (b) AuNP@PCN-222(Cu). (c) PCN-222(Cu).

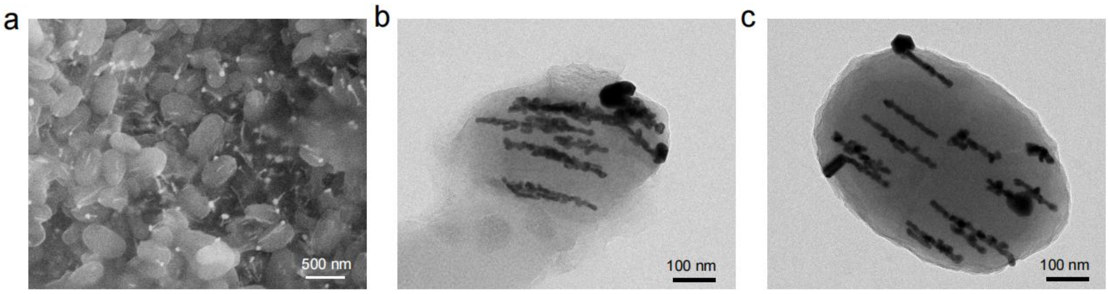

Supplementary Fig. 13. (a) SEM image of AuNN@PCN222(Cu) after ten hours of electrolysis at -1.2 V vs RHE. (b) TEM image of a partially disintegrated AuNN@PCN222(Cu) after the 10-h CO_2RR test. (c) TEM image of an intact AuNN@PCN222(Cu) after the 10-h CO_2RR test.

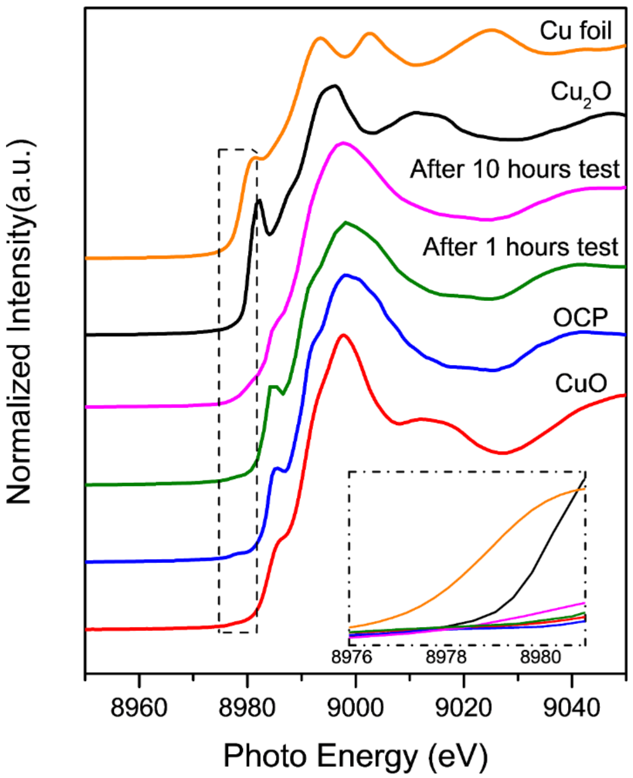

Supplementary Fig. 14. Cu K-edge XANES spectra taken on AuNN@PCN-222(Cu) after different reaction time.

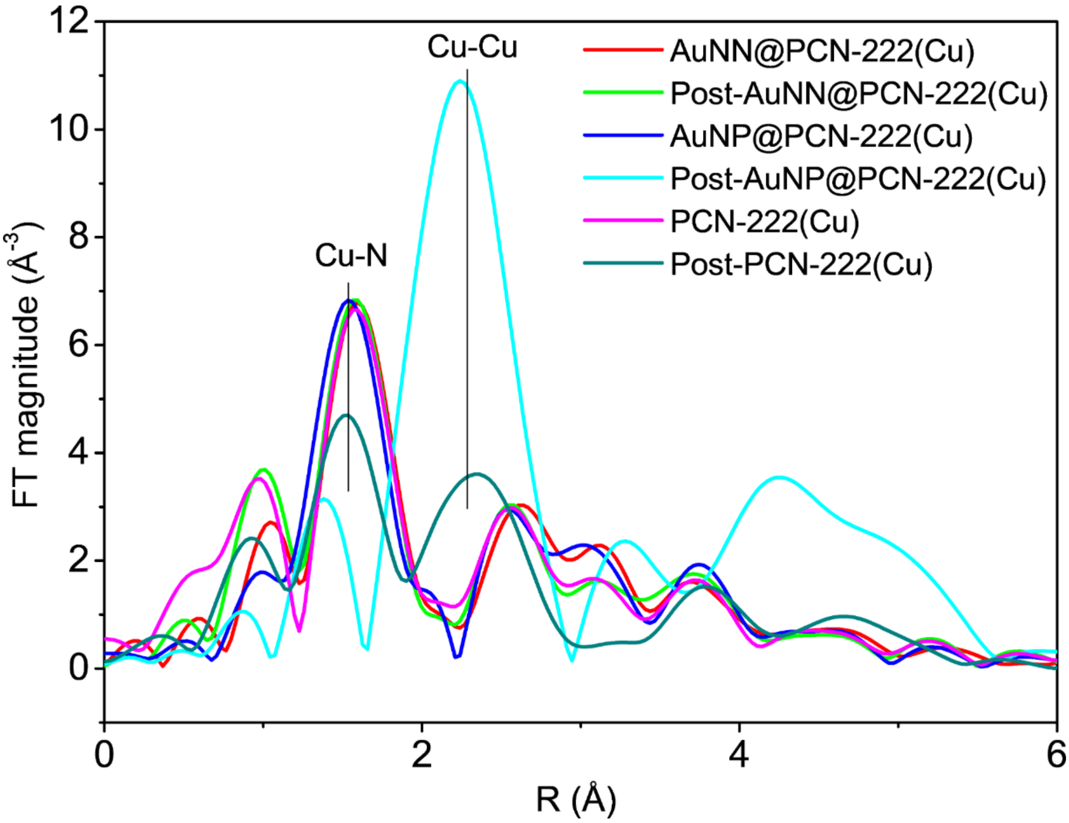

Supplementary Fig. 15. Fourier-transform extended X-ray absorption fine structure of AuNN@PCN-222(Cu), AuNP@PCN-222(Cu) and PCN-222(Cu) before and after 1 h CO_2RR test at -1.2 V.

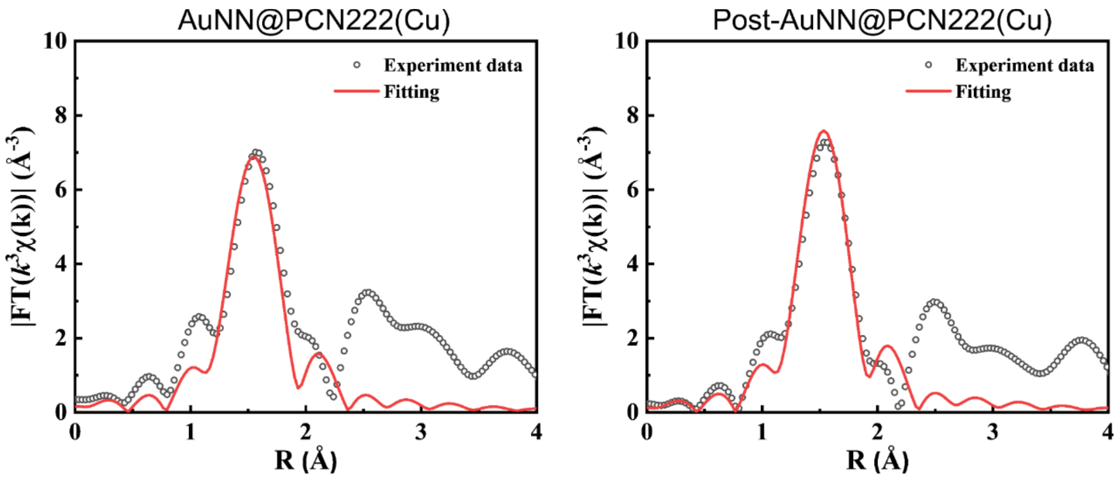

Supplementary Fig. 16. Fitting of the Cu K-edge EXAFS spectra for AuNN@PCN-222(Cu) before and after 1 h CO_2RR test at -1.2 V. See Table S3 for fitting details.

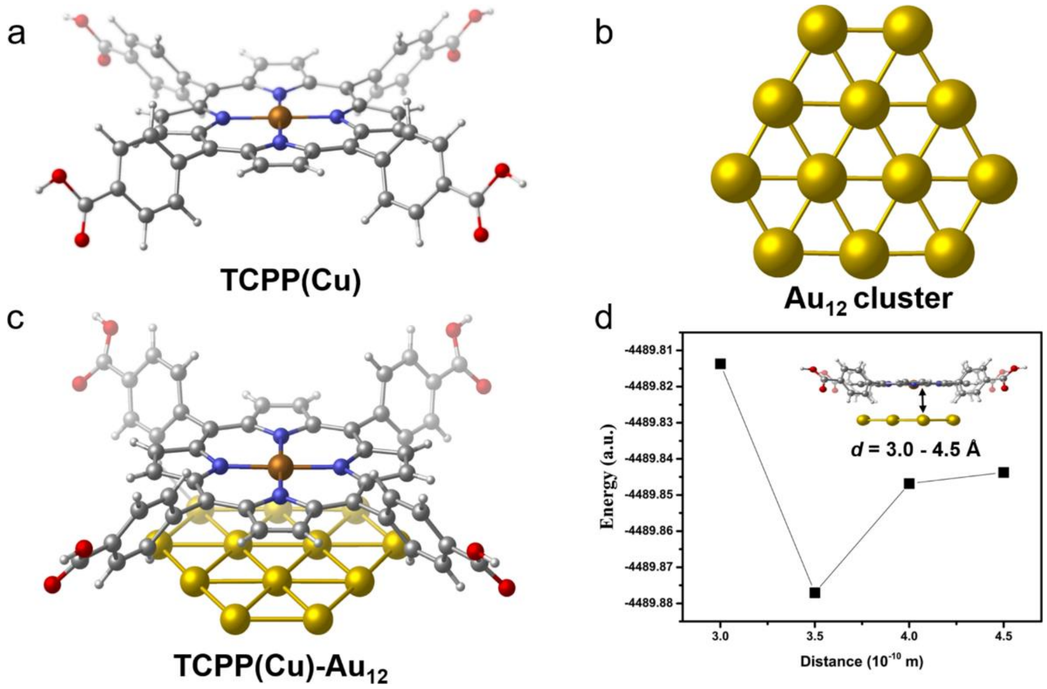

Supplementary Fig. 17. (a-c) Structural model of TCPP(Cu)-Au_{12}. (d) Free energies of TCPP(Cu)-Au_{12} by modulating the TCPP(Cu)/Au_{12} distance. The colors are: C in silver, H in white, O in red, N in blue, and Cu in orange.

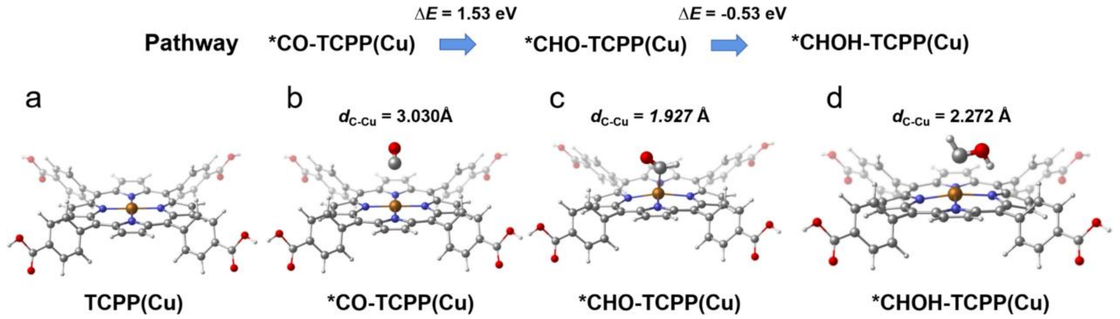

Supplementary Fig. 18. Configuration of intermediates binding on TCPP(Cu). (a) Stand-alone, (b) *CO, (c) *CHO, and (d) *CHOH.

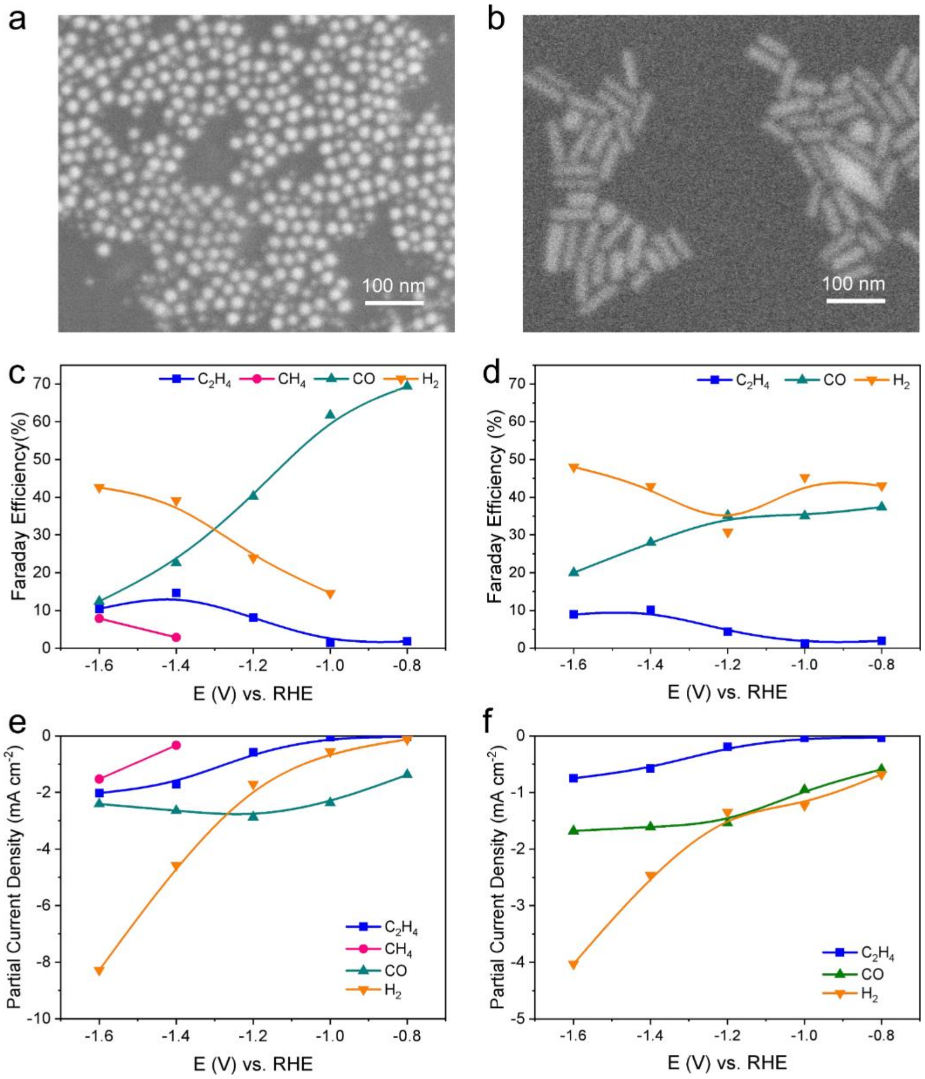

Supplementary Fig. 19. (a, b) SEM images of Au nanoparticles and Au nanorods, respectively. (c, d) Product FEs at different potentials for Au-nanoparticles@TCPP(Cu) and Au-nanorods@TCPP(Cu), respectively. (e, f) Partial current densities of various reduction products for Au-nanoparticles@TCPP(Cu) and Au-nanorods@TCPP(Cu), respectively.

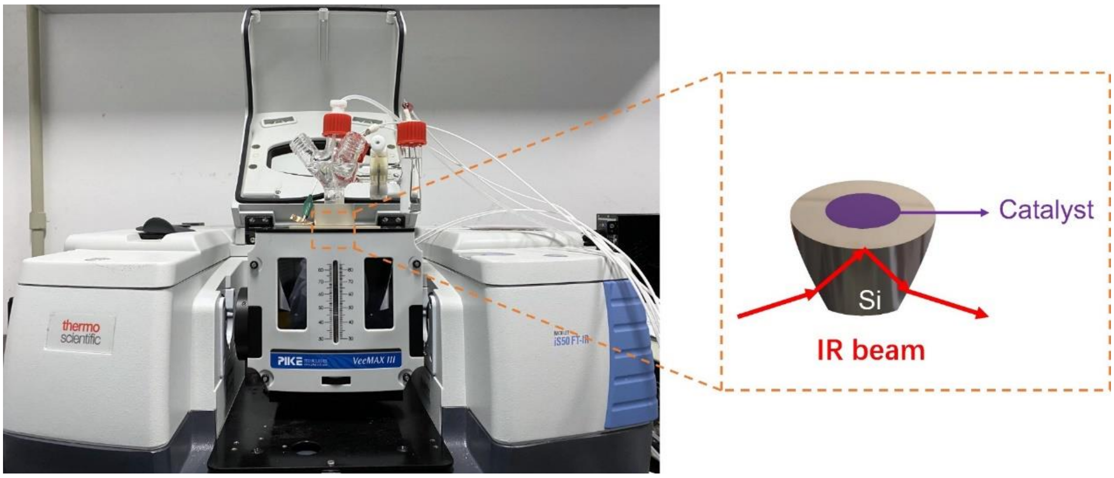

Supplementary Fig. 20. Schematic setup and photograph of the ATR-SEIRAS used for CO_2RR.

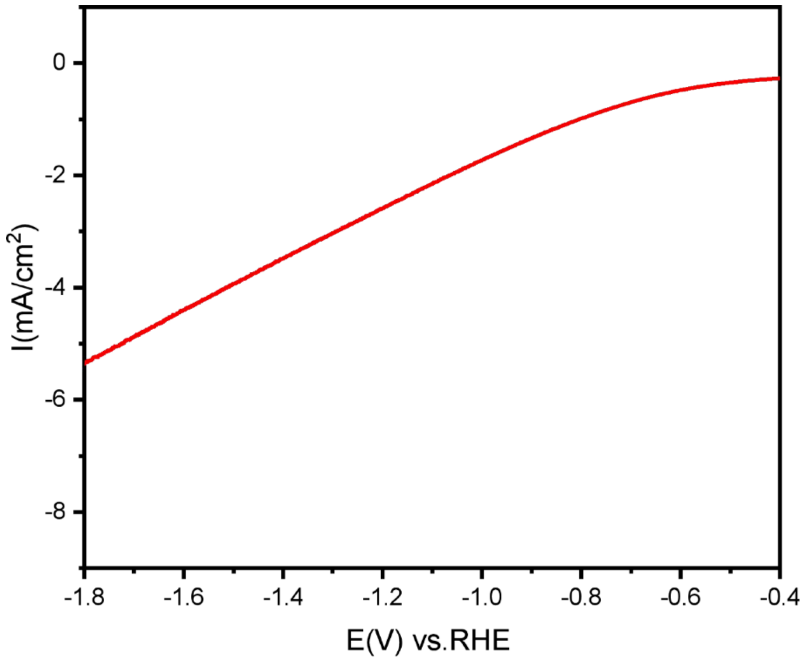

Supplementary Note 3： LSV of AuNN@PCN222(Cu) in Operando ATR-SEIRAS

Note that the internal resistance of the spectro-electrochemical cell was much higher than that of the regular H cell, so that the applied potential was swept from -0.4 to 1.8 V.

Supplementary Table 1. Elemental analysis of the catalysts by Inductively Coupled Plasma - Emission Spectrometry (ICP-ES)

	**Zr**	**Cu**	**Au**
**PCN222(Cu)**	10%	3.5%	0
**Au-NN@PCN222(Cu)**	9.4%	3.4%	7.1%
**Au-NP@PCN222(Cu)**	9.3%	3.3%	7.3%

Supplementary Table 2. Comparison of AuNN@PCN-222(Cu) with previously reported MOF electrocatalysts on CO_2RR performances.

Catalyst	Main product (FE, E vs RHE)	Stability	Active sites	reference
Au-NN@PCN-222(Cu)	C_2H_4 (52.5% at -1.2 V)	10 h	Au/Cu-N_4	This work
PML-Cu	$HCOO^-$ (80.86% at -0.7 V)	5 h	Cu-O_4	1
CPF	CH_4 (56% at -1.4 V)<br>C_2H_4 (32% at -1.1 V)	2 h	Cu-porphyrin	2
Cu_2BDC	C_2H_4 + C_2H_5OH (46.8% at -1.3 V)		Cu_2O & Cu	3
NNU-33(H)	CH_4 (82% at -0.9 V)	5 h	Cu(I)-Cu(I)	4
PcCu-Cu-O	C_2H_4 (50% at -1.2 V)	4 h	PcCu-CuO_4	5
Cu-THQ	CO (91% at -0.45 V)		Cu-O	6
HKUST-1	C_2H_4 (45% at -1.07 V)	7.5 h	Cu cluster	7
CuPc	CH_4 (66% at -1.06 V)		Cu cluster	8

Supplementary Table 3. Fitting results of the FT-EXAFS spectra in Supplementary Fig. 15

**Sample**	**Path**	**CN[a]**	**R(Å)[b]**	**ΔE_0(eV)[c]**	**σ^2(10^{-3} Å^2)[d]**
AuNN@PCN-222(Cu)	Cu-N	3.9	1.99	-0.01	3.40
Post-AuNN@PCN-222(Cu)	Cu-N	4.0	1.97	-0.01	2.65

S_0^2 was set to 0.85, according to the experimental EXAFS fitting of Cu foil by fixing CN as the known crystallographic values. a CN is the coordination number; [b] R is interatomic distance (the bond length between central atoms and surrounding coordination atoms); [c] ΔE_0 is edge-energy shift (the difference between the zero kinetic energy value of the sample and that of the theoretical model); [d] σ^2 is Debye-Waller factor (a measure of thermal and static disorder in absorber-scatterer distances).

Supplementary Table 4. Mulliken Charge analysis (Q(e)) for the adsorbed intermediates and Cu and N atoms in the TCPP(Cu) and TCPP(Cu)-Au_{12} system

		$Q_{(e)}$ (Mulliken)									
**System**		Cu	N_1	N_2	N_3	N_4					
**TCPP(Cu)**	**TCPP(Cu)**	0.688	-0.649	-0.649	-0.649	-0.649					
		Cu	N_1	N_2	N_3	N_4	*C	O			
	***CO**	0.585	-0.646	-0.646	-0.641	-0.640	0.274	-0.189			
		Cu	N_1	N_2	N_3	N_4	*C	H	O		
	***CHO**	0.167	-0.611	-0.552	-0.610	-0.587	0.385	0.309	-0.297		
		Cu	N_1	N_2	N_3	N_4	*C	H	O	H	
	***CHOH**	0.392	-0.621	-0.693	-0.621	-0.627	0.084	0.192	-0.512	0.541	
**TCPP(Cu)-Au_{12}**		Cu	N_1	N_2	N_3	N_4					
	**TCPP(Cu)-Au_{12}**	0.650	-0.628	-0.613	-0.618	-0.612					
		Cu	N_1	N_2	N_3	N_4	*C	O			
	***CO**	0.606	-0.624	-0.610	-0.616	-0.610	0.294	-0.188			
		Cu	*N_1	N_2	N_3	N_4	*C	H	O		
	***CHO**	0.420	-0.584	-0.564	-0.581	-0.567	0.387	0.269	-0.429		
		Cu	*N_1	*N_2	N_3	N_4	*C	H	O	*C	O
	***CHO +*CO**	0.378	-0.601	-0.600	-0.590	-0.584	0.391	0.279	-0.420	0.266	-0.178
		Cu	N_1	N_2	N_3	N_4	*C	O	C	H	O
	***CO-CHO**	0.270	-0.573	-0.576	-0.564	-0.558	0.547	-0.316	0.234	0.287	-0.365

Supplementary Table 5. The adsorption energy (ΔEads, in eV) of *CO and *CHO on TCPP(Cu) and TCPP(Cu)-Au_{12}

**System**		***ΔE_{ads}* (eV)**
TCPP(Cu)	*CO	-0.186
	*CHO	0.375
TCPP(Cu)-Au_{12}	*CO	-0.269
	*CHO	-0.746

Here, $\Delta E_{ads} = E_{intermediate\text{-}substrate} - (E_{intermediate} + E_{substrate})$, more negative value stands for stronger binding. We see that the incorporation of Au substrate dramatically enhances CHO binding.

References

1. Yang, D.; Zuo, S.; Yang, H.; Zhou, Y.; Wang, X., Freestanding Millimeter-Scale Porphyrin-Based Monoatomic Layers with 0.28 nm Thickness for CO_2 Electrocatalysis. *Angew Chem Int Ed Engl* **2020,** *59* (43), 18954-18959.

2. Zhou, Y.; Chen, S.; Xi, S.; Wang, Z.; Deng, P.; Yang, F.; Han, Y.; Pang, Y.; Xia, B. Y., Spatial Confinement in Copper-Porphyrin Frameworks Enhances Carbon Dioxide Reduction to Hydrocarbons. *Cell Reports Physical Science* **2020,** *1* (9), 100182.

3. Zhou, X.; Dong, J.; Zhu, Y.; Liu, L.; Jiao, Y.; Li, H.; Han, Y.; Davey, K.; Xu, Q.; Zheng, Y.; Qiao, S. Z., Molecular Scalpel to Chemically Cleave Metal-Organic Frameworks for Induced Phase Transition. *J Am Chem Soc* **2021,** *143* (17), 6681-6690.

4. Zhang, L.; Li, X. X.; Lang, Z. L.; Liu, Y.; Liu, J.; Yuan, L.; Lu, W. Y.; Xia, Y. S.; Dong, L. Z.; Yuan, D. Q.; Lan, Y. Q., Enhanced Cuprophilic Interactions in Crystalline Catalysts Facilitate the Highly Selective Electroreduction of CO_2 to CH_4. *J Am Chem Soc* **2021,** *143* (10), 3808-3816.

5. Qiu, X. F.; Zhu, H. L.; Huang, J. R.; Liao, P. Q.; Chen, X. M., Highly Selective CO_2 Electroreduction to C2H4 Using a Metal-Organic Framework with Dual Active Sites. *J Am Chem Soc* **2021,** *143* (19), 7242-7246.

6. Majidi, L.; Ahmadiparidari, A.; Shan, N.; Misal, S. N.; Kumar, K.; Huang, Z.; Rastegar, S.; Hemmat, Z.; Zou, X.; Zapol, P.; Cabana, J.; Curtiss, L. A.; Salehi-Khojin, A., 2D Copper Tetrahydroxyquinone Conductive Metal-Organic Framework for Selective CO_2 Electrocatalysis at Low Overpotentials. *Adv Mater* **2021,** *33* (10), e2004393.

7. Nam, D. H.; Bushuyev, O. S.; Li, J.; De Luna, P.; Seifitokaldani, A.; Dinh, C. T.; Garcia de Arquer, F. P.; Wang, Y.; Liang, Z.; Proppe, A. H.; Tan, C. S.; Todorovic, P.; Shekhah, O.; Gabardo, C. M.; Jo, J. W.; Choi, J.; Choi, M. J.; Baek, S. W.; Kim, J.; Sinton, D.; Kelley, S. O.; Eddaoudi, M.; Sargent, E. H., Metal-Organic Frameworks Mediate Cu Coordination for Selective CO_2 Electroreduction. *J Am Chem Soc* **2018,** *140* (36), 11378-11386.

8. Weng, Z.; Wu, Y.; Wang, M.; Jiang, J.; Yang, K.; Huo, S.; Wang, X. F.; Ma, Q.; Brudvig, G. W.; Batista, V. S.; Liang, Y.; Feng, Z.; Wang, H., Active sites of copper-complex catalytic materials for electrochemical carbon dioxide reduction. *Nat Commun* **2018,** *9* (1), 415.
